# Microfluidic device for enhancement and analysis of osteoblast differentiation in three-dimensional cell cultures

**DOI:** 10.1186/s13036-023-00395-z

**Published:** 2023-12-14

**Authors:** Michael Killinger, Adéla Kratochvilová, Eva Ingeborg Reihs, Eva Matalová, Karel Klepárník, Mario Rothbauer

**Affiliations:** 1 Department of Bioanalytical Instrumentation, Institute of Analytical Chemistry, Academy of Sciences, Brno, Czech Republic; 2 Department of Chemistry, Faculty of Science, Masaryk University, Brno, Czech Republic; 3 Laboratory of Odontogenesis and Osteogenesis, Institute of Animal Physiology and Genetics, Academy of Sciences, Brno, Czech Republic; 4 Cell Chip Group, Institute of Applied Synthetic Chemistry, Institute of Chemical Technologies and Analytics, Faculty of Technical Chemistry, Technical University Vienna, Vienna, Austria; 5 Karl Chiari Lab for Orthopaedic Biology, Department of Orthopedics and Trauma Surgery, Medical University of Vienna, Vienna, Austria

**Keywords:** Bone-on-a-chip, 3D cell cultures, Dynamic cultivation, Microfluidics, Microwells micropillars

## Abstract

Three-dimensional (3D) cell cultures are to date the gold standard in biomedical research fields due to their enhanced biological functions compared to conventional two-dimensional (2D) cultures. 3D cell spheroids, as well as organoids, are better suited to replicate tissue functions, which enables their use both as in vitro models for basic research and toxicology, as well as building blocks used in tissue/organ biofabrication approaches. Culturing 3D spheroids from bone-derived cells is an emerging technology for both disease modelling and drug screening applications. Bone tissue models are mainly limited by the implementation of sophisticated devices and procedures that can foster a tissue-specific 3D cell microenvironment along with a dynamic cultivation regime. In this study, we consequently developed, optimized and characterized an advanced perfused microfluidic platform to improve the reliability of 3D bone cell cultivation and to enhance aspects of bone tissue maturation in vitro. Moreover, biomechanical stimulation generated by fluid flow inside the arrayed chamber, was used to mimic a more dynamic cell environment emulating a highly vascularized bone we expected to improve the osteogenic 3D microenvironment in the developed multifunctional spheroid-array platform. The optimized 3D cell culture protocols in our murine bone-on-a-chip spheroid model exhibited increased mineralization and viability compared to static conditions. As a proof-of-concept, we successfully confirmed on the beneficial effects of a dynamic culture environment on osteogenesis and used our platform for analysis of bone-derived spheroids produced from primary human pre-osteoblasts. To conclude, the newly developed system represents a powerful tool for studying human bone patho/physiology in vitro under more relevant and dynamic culture conditions converging the advantages of microfluidic platforms with multi-spheroid array technologies.

**Graphical Abstract:**

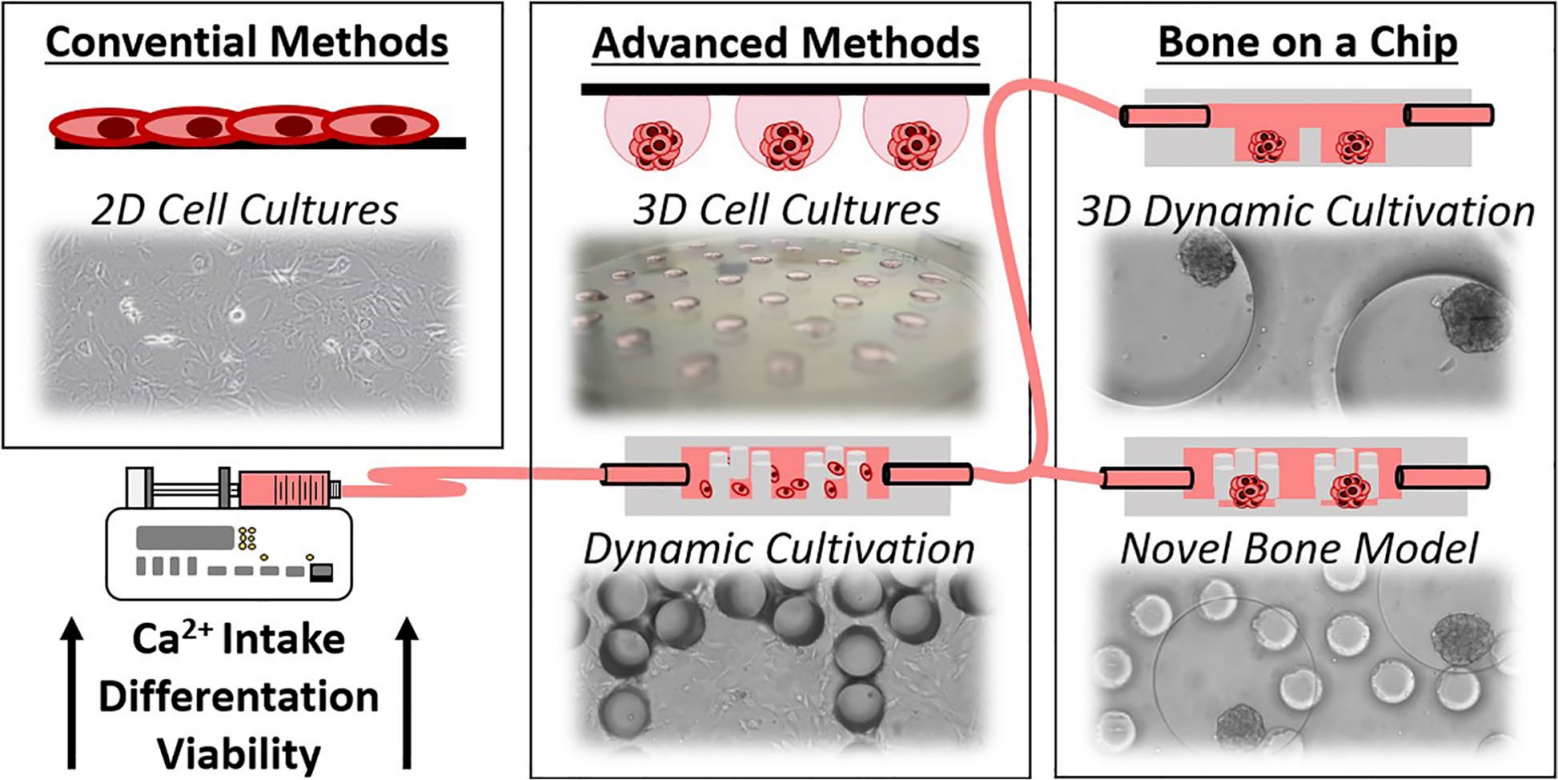

**Supplementary Information:**

The online version contains supplementary material available at 10.1186/s13036-023-00395-z.

## Background

In vitro bone formation assays and disease modelling are still mostly performed using two-dimensional (2D) culture systems to investigate the chemical differentiation of osteoblast precursors into osteoblasts (OBs) and potentially also osteocytes. However, these culturing methods do not reliably simulate in vivo bone formation. Recently, 3D cell culture systems have received attention as a powerful tool for bone translation medicine to gather a better glance into osteogenic processes and to properly mimic the in vivo situation [1]. Moreover, 3D formats of osteogenic precursors provide a more realistic and representative model of the tissue’s natural microenvironment. The 3D structures also exhibited notably upregulated and downregulated OB marker levels with the secretion of bone-specific extracellular matrix (ECM) components compared to those of conventional 2D monolayers [1–3].

Mineralization and ECM deposition can also potentially be enhanced by the cultivation of cells in a dynamic flow of media mimicking in vivo vascularization. The process of vascularization is crucial not only for continuous perfusion of nutrients and removal of cellular waste metabolites but also for inducing fluid mechanical stimulation, which plays an important role in osteogenesis and bone regeneration [4]. The efficient biomimicry of mechanisms of vascularized bone with 3D cell structures in vitro is still a major scientific and technical challenge since larger 3D constructs require a constant nutrient supply to survive in vitro. A promising tool for increasing the sustainability of 3D cell structures is the more recent application of microfluidic bone biochips to control dynamic cell culture development. Microfluidic technology allows precise control over general culture conditions, automatization, as well as reduced amounts of reagents and cells. More importantly to bone bioengineering, microfluidics enable dynamic conditions for continuous perfusion of nutrients and removal of the metabolic waste emulating a more biomimetic environment found in actual bone tissue. Notably, the flow of media additionally serves as a potent stimulant exposing the cells to shear stress similar to the actual dynamic niche found to retain bone cell physiology. The fluid shear stress generated by fluid flow inside the lacunar-canalicular networks and trabecular spaces within bone tissue is important for the maintenance of skeletal tissue architecture and homeostasis.

Here, we describe the design, fabrication, optimization, and characterization of a newly developed miniaturized microfluidic screening platform technology for the dynamic cultivation of osteoblast-derived bone spheroid cultures. We detail for the first time the development and assessment of a microscopy-enabled benchtop platform for on-chip osteogenesis that provides a reproducible investigation of multiple 3D bone cell cultures under dynamic flow conditions using a combination of microfluidic channels with microcavity arrays. Initially, we optimized the cultivation protocols for 3D bone cell formation and cultivation of murine MC3T3-E1 pre-osteoblasts. Several methods were tested, including hanging drops, ECM gels and even anti-adhesive surface coatings to optimise the general approaches to spheroid formation. The condensation and maturation of spheroids of murine pre-osteoblasts and primary human bone-derived osteoblasts led to the increase of osteogenic differentiation markers. Dynamic cultivation increased Ca^2+^ intake of 2D cell cultures as well as the viability of 3D cell murine cultures, thus representing a valid microsystems approach to study bone biology with multi-plexed spheroids. In summary, we developed an optimized microwell system suitable for multi-spheroid generation with micropillar technologies set out to mimic the basic structure of a trabecular bone environment to potentiate osteogenic potential, which was confirmed for on-chip differentiation and maintenance of human bone-derived spheroids under dynamic 3D culture conditions.

## Results

### Establishment of a miniaturized optically accessible microsystems platform technology

In this study, we detail for the first time the design, development, and assessment of a microfluidic osteogenesis-on-a-chip device that incorporates 3D bone cell cultures and dynamic microfluidic conditions as stand alone platform technology operated on a conventional microscope set-up. The initial development strategy was defined to result in (i) a technical solution for the establishment of a miniaturized autonomous incubator with microfluidic cultivating inserts that can be easily fabricated according to requirements and can be designed for a variety of applications, and (ii) the optimization of multi-plexed 3D bone spheroid models for validation of the positive effect of dynamic cultivation to support the differentiation of 3D osteoblast cultures while reducing the disadvantages of spheroid-based cultivation systems such as necrotic core formation.

**Fig. 1:**
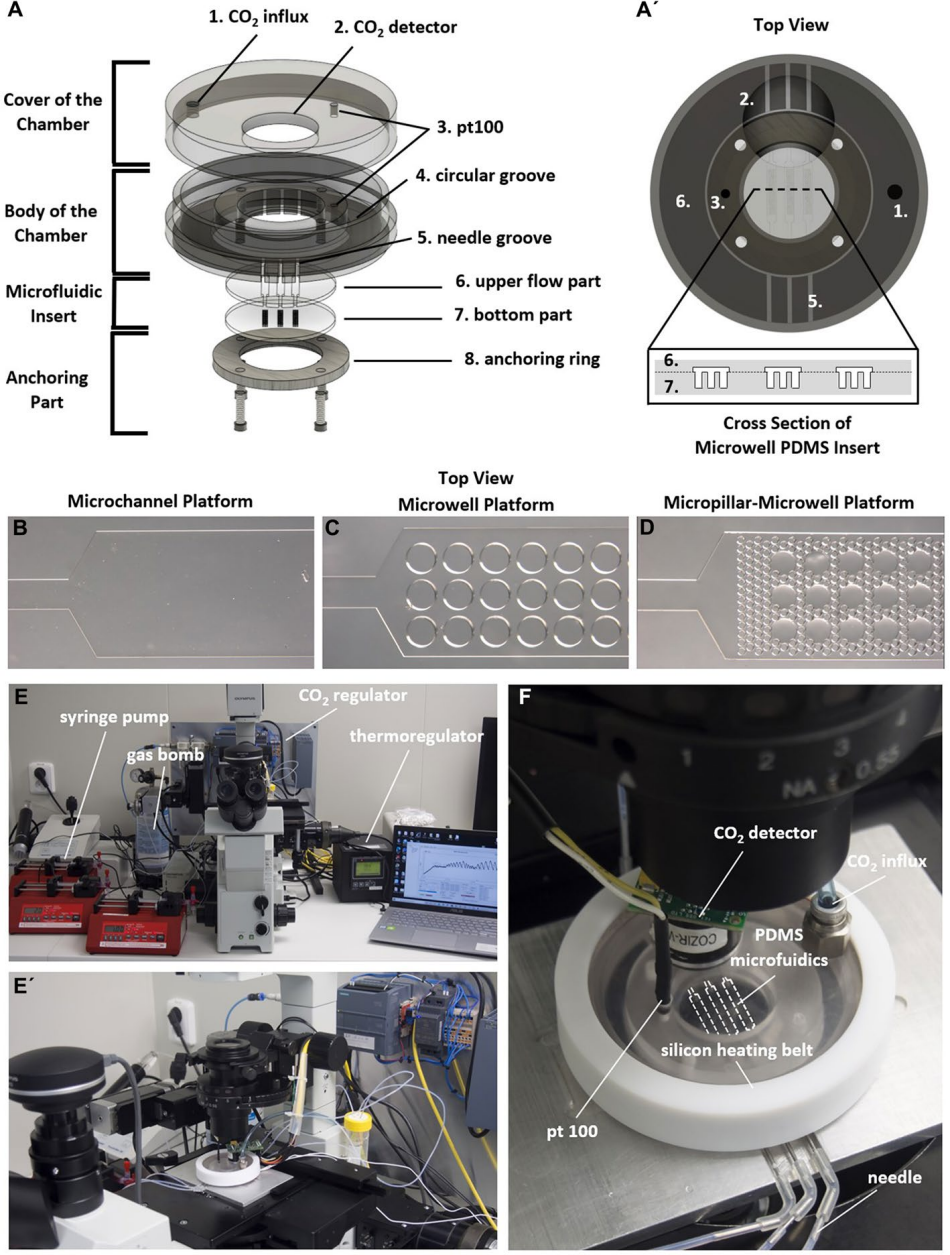
Design of the miniaturized, microfluidic, and optically accessible chamber. **A** Assembly parts with holes for inserts (1–5). **A´** Top view of cultivation chamber with cross section of PDMS microwell platform. Microwell platform include (6.) media flow part and (7.) microwell part. The microfluidic inserts are anchored by a ring and four screws (8.). **B**-**D** Different PDMS microfluidics inside the culture chamber. **E**, **E´** Real prototype of the miniaturized autonomous chamber with control of temperature and CO_2_ concentration placed on a microscope table for continual observation. **F** Detailed image of the mounted microfluidic spheroid platform comprising a microfluidic insert, a silicon heating belt, a pt100 temperature sensor and a CO_2_ sensor for precise control of the cultivation environment

We prototyped a miniaturized incubator with autonomous CO_2_ control and integrated thermoregulation (Fig. 1). The incubator was composed of a cover, the body of the chamber, a PDMS microfluidic insert and an anchoring ring (Fig. 1A, A´). Here, we tested several microfluidic inserts for cell cultivation to optimise the design and conditions for 3D cell formation and subsequent cultivation including three distinct microfluidic strategies termed microchannel, microwell and micropillar approach. Initially, a primitive microchannel platform was fabricated (Fig. 1B). The microchannel platform was used to test the physical stability and function of our designed miniaturized incubator system as well as the effect of a dynamic conditions on conventional 2D murine pre-osteoblast cell cultures to act as controls for different platforms for 3D bone formation. Microwell platforms (Fig. 1C) are well-established platforms for 3D cell culture fabrication and cultivation, but the media flow is restricted just to the top part of the microwell, while spheroids inside microcavities may be subjected to irregular and uncontrollable fluid flows. To improve flow profiles and to potentially approximate the basic design towards a niche that resembles trabecular bone, we designed a micropillar platform (see Supp. Chap. 3, 5) and a platform combining microwells with micropillars (Fig. 1D). The whole microfluidic assembly was connected for fluid flow adjustment to a syringe pump and placed on a microscope table for online monitoring (Fig. 1E, E´). To ensure a reproducible cell culture environment outside conventional incubators, the platforms CO_2_ concentration and temperature were precisely monitored and regulated by an autonomous system (Fig. 1F).

### Murine pre-osteoblasts cultivated under dynamic conditions exhibited increased mineralization and more dendritic morphology

**Fig. 2:**
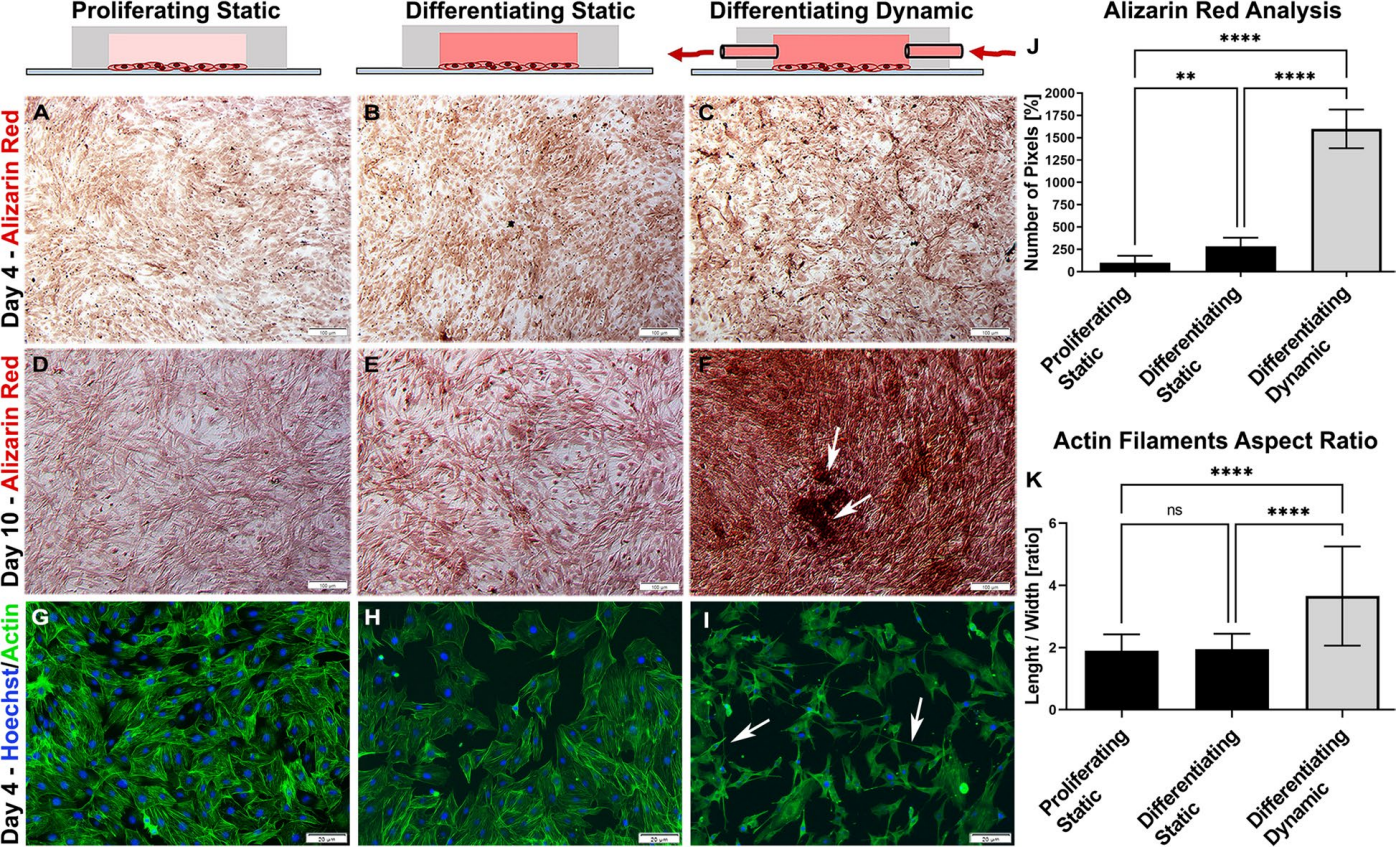
Impact of dynamic culture conditions on the osteogenic differentiation of 2D on-chip cultures of MC3T2-E1 cells. Osteogenic differentiation capacity (Alizarin red staining) of cells cultivated in the 2D microfluidic biochip system for 4 (**A**-**C**) and 10 days (**D**-**F**). Significant increased Ca^2+^ intake was observed in the cells cultivated in differentiation media in static (**E**, 284% ± 97, *p* = 0.0029) and dynamic (**F**, 1600% ± 217, *p* < 0.0001) conditions using image analysis. Cells cultivated under dynamic conditions also generated micromass formations (**F**-arrows). **G**-**I** Cell spreading analysis using fluorescence staining (blue: Hoechst 33,342; green: **F**-actin). Cultivation of the cells under dynamic conditions for 4 days led to dendrite formation (**I**; white arrows) and exhibited a significantly higher aspect ratio (length vs. width depicted in K). This ratio was established as 1.9 ± 0.5, *p* = 0.9355 for proliferating cells, 1.9 ± 0.5, *p* < 0.0001 for cells cultivated in differentiation media and 3.6 ± 1.5 *p* < 0.0001 for cells cultivated in dynamic conditions (**K**). Scale bars = 100 μm (brightfield images) and 20 μm (fluorescence images)

Osteoblast differentiation is related to increased extracellular matrix mineralization. To validate the positive effect of our on-chip planar cultivation approach as outlined in the previous chapter, the cells were initially seeded at a concentration of 10^5^ cells/ml and cultivated inside microchannel units of the PDMS insert module. After 4 and 10 days of cultivation, the cells were fixed and stained with Alizarin red to reveal the level of mineralization. The cells cultivated in the microcultivation chamber exhibited opticaly higher levels of Ca^2+^ deposition within 4 days of cultivation (Fig. 2A-C). The level of mineralized extracellular matrix under static conditions was lower than that under dynamic conditions (Fig. 2C) in both proliferation (Fig. 2A) and differentiation media (Fig. 2B). The intensity of Ca^2+^ staining also increased during cultivation (Fig. 2D-F) showing a gradual increase in the mineralization of osteoblastic cells when cultivated on-chip under static conditions by threefold (284 ± 97%) and dynamic differentiation media by fifteen-fold (1600 ± 217%) when compared to cells cultured in static conditions using only proliferating media (100 ± 79%) (Fig. 2J). Moreover, cultivation under dynamic conditions led to the formation of micromass condensation spots, which is known to further boost osteogenic mineralization in the condensation core of conventional bone models, also highlighting the capacity of MC3T3-E1 cells to form such enhanced 3D cell culture characteristics under dynamic conditions (white arrow indicators shown in Fig. 2F).

The so-called osteoblast-osteocyte switch is related to changes in cell morphology, polarization, gene expression, and extracellular matrix deposition as well as occurrence of cell dendrites or pseudopodia during differentiation [5]. To observe such early changes, we next stained the actin filaments after 4 days of cultivation under dynamic conditions and again compared these intricate structural differences to morphological characteristics of the static cultivation controls. Cells were either cultivated in static conditions using proliferation (Fig. 2G) or differentiation media (Fig. 2H) in triplicate for 4 days. Dynamic cultivation was performed in a miniaturized chamber with autonomous CO_2_ and temperature regulation in three independent microchannels. After initial seeding and adhesion under static conditions, the overnight differentiation medium was pumped at a flow rate of 2 µl/min to each individual cultivation unit for the next 3 days. The results of this experiment suggest that the cells cultivated under dynamic conditions exhibited longer protrusions and displayed thinner morphology in contrast to cells cultivated in static conditions (Fig. 2I, white arrows). Additional image analysis revealed a significantly higher aspect ratio (length vs. width) compared to the cells cultivated under static conditions (Fig. 2K). This effect of media flow force on osteoblast cell spreading is in line with previous studies [6].

### The on-chip dynamic 3D culture environment can better induce osteoblast differentiation

**Fig. 3:**
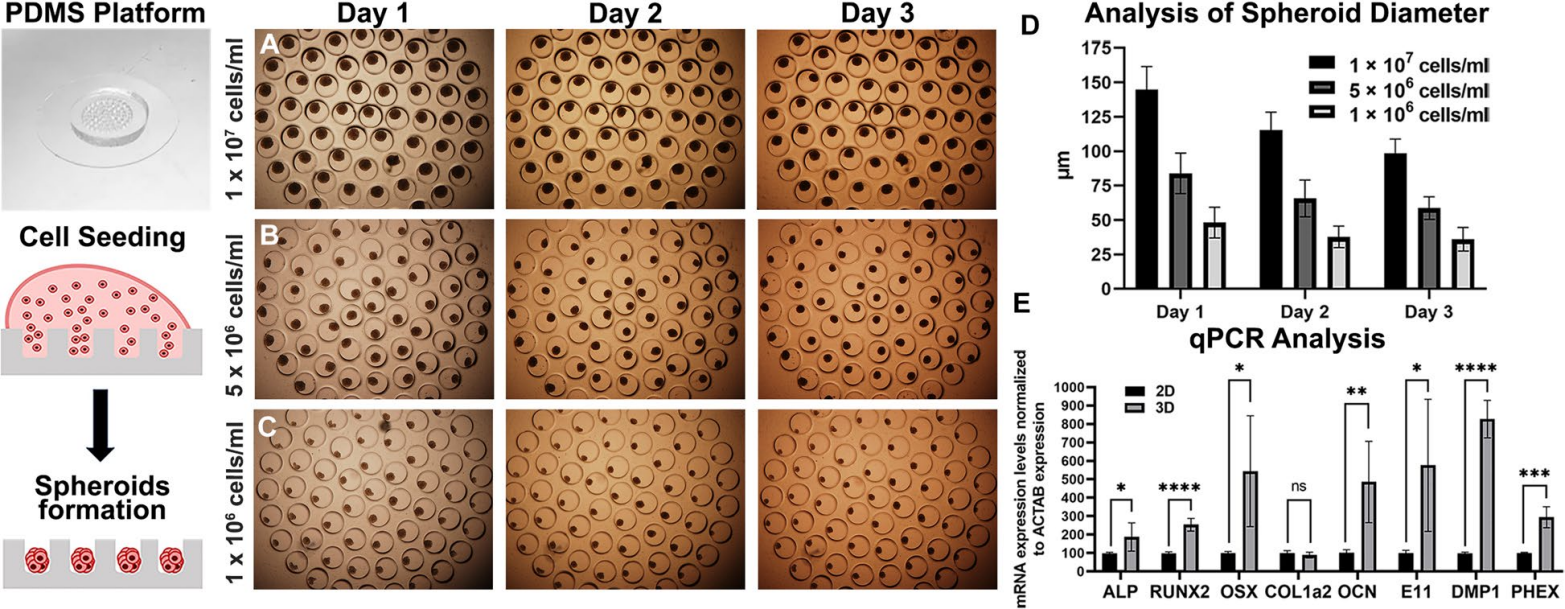
**A**-**C** Spheroid formation using the PDMS microwell platform and (**D**) analysis of spheroid diameters at different initial seeding densities during 3 days of cultivation (*n* = 60). **E** Actin-normalized mRNA expression levels of osteoprogenitor, preosteoblast, and osteoblast markers in the monolayer and spheroids were measured by qPCR. *n* ≥ 3, * *p* < 0.05, ** *p* = 0.0019, *** *p* = 0.0008, **** *p* < 0.0001. Scale bar = 100 μm, microwell spheroids were captured under 40x magnification

To test whether the differentiation capacity can be further enhanced, we next established a microfluidic cultivation protocol for generating bone spheroids. The formation and maturation of MC3T3-E1 cells as spheroids in an advanced nonperfused multiwell platform was optimized and benchmarked against frequently used hanging drop cultivation approaches, that represent the most established technique to investigate primary cell cultures in a advanced 3D environment. To investigate the osteogenic differentiation capacity of MC3T3-E1 spheroids under potentially more reproducible culture conditions, we next fabricated a PDMS microwell platform and investigated the effect of microwell spheroid generation on osteogenic differentiation capacity using qPCR analysis (see Fig. 3A-C). A major advantage of this approach is that in contrast to manual handling with hanging drops and hydrogel-based protocols that are highly variable, spheroids inside a miniaturized platform are less prone to user variability as well as mechanical damage during media replacement while providing multiple technical spheroid replicates at a time from a single injection volume. In detail, we developed a PDMS microwell platform containing 64 microwells with a diameter of 400 μm, a space of 100 μm, and a height of 400 μm. The multi-spheroid inserts were coated with a Pluronic F-127 as anti-adhesive surface coating to prevent cell adhesion and anchored by force to 48-well plates or Petri dishes. The cell suspension was placed in 30 µl drops on the surface of the microwell at initial cell concentrations of 1 × 10^6^, 5 × 10^6^, and 1 × 10^7^ cells per milliliter (Fig. 3C-E). The different concentrations led to the formation of spheroids of diverse sizes and decreased gradually in mass during the cultivation period (Fig. 3D). At the initial cell seeding concentration of 1 × 10^7^ cells per milliliter, the initial size of 144 μm (± 17) at day 1 decreased to 115 μm (± 13 μm difference) and 98 μm (± 11 μm difference) for day 2 and 3, respectively. The initial seeding concentration 5 × 10^6^ led to the formation of 84.0 μm (± 15) spheroids with the decreasing size during 3 day cultivation to 66 μm (± 13 μm difference) after 2 and 59 μm (± 9 μm difference) 3 days of cultivation. At the lowest concentration 1 × 10^6^, the size of the spheroids was 49 μm (± 11) during the first day of cultivation, 39 μm (± 7 μm difference) the second day and 37 μm (± 8 μm difference) the third day of cultivation. Even though a linear spheroid size decrease was observed also for lower seeding densities, the overall shrinking effect was slightly reduced for the lowest seeding density compared to the highest with 25% and 32%, respectively.

Next, as shown in Fig. 3E, the gene expression levels were examined via qPCR for osteoprogenitor, preosteoblast, and osteoblast markers comparing the monolayer and microwell spheroids of the 1 × 10^7^ cells per milliliter seeding density. Our results indicated an overall improvement of osteogenic gene expression with multiplexed bone spheroids with expression of RUNX2 (254 ± 34%, *p* < 0.0001), ALP (188 ± 76%, *p* = 0.0316), COL1a1 (90 ± 15%, ns), OCN 486 ± 220%, *p* = 0.0019), OSX (544 ± 300%, *p* = 0.0280), PHEX (295 ± 56%, *p* = 0.0008), DMP1 (827 ± 102%, *p* < 0.0001) and E11 (577 ± 358%, *p* = 0.0375). Notably, the expression levels of all the osteogenic marker genes with the exception of COL1a1 were increased indicating at an improved osteogenic environment in our microwell platform.

**Fig. 4:**
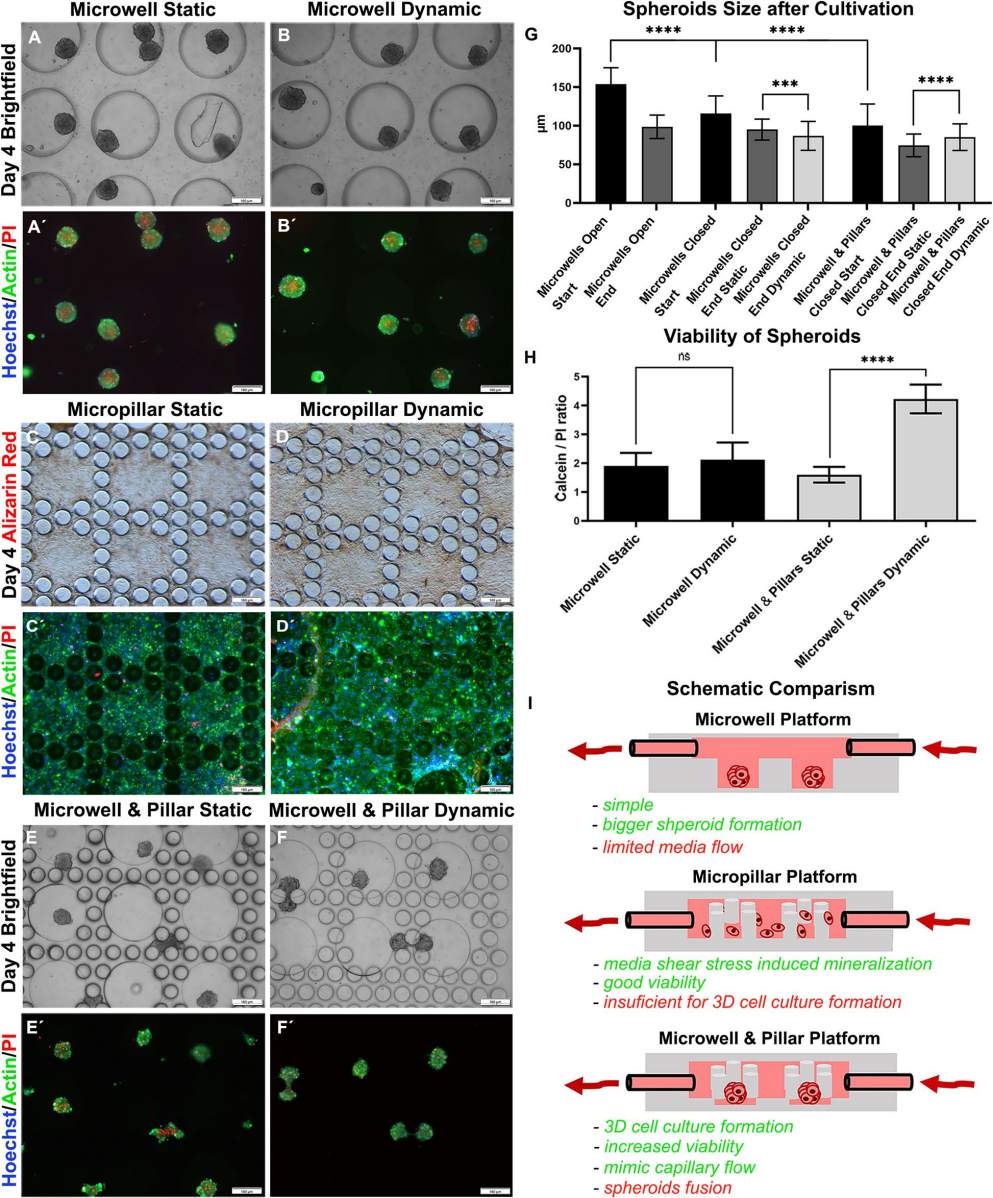
Comparison of microfluidic cultivation platforms after 4 days of cultivation. Spheroids cultivated in the microwell (**A**, **B**) platform in the cultivation chamber were smaller than spheroids cultivated in the open microwell platform (**G**). Analysis of mineralization (**C**, **D**) and viability (**C**´, **D**´) of MCT3T3-E1 cells in micropillar microfluidics, which was not sufficient for spheroid formation. (**E**, **F**) Spheroid formation in the microwell-pillar platform. The spheroids cultivated inside the microwell-pillar platform exhibited an altered decrease in sphere size (**G**), and propidium iodide staining showed the positive effect of dynamic cultivation on spheroids (**H**). **I** Summarizing scheme. **G** *n *= 120, *** *p*<0005, **** *p*<00001. (H) *n *= 30, **** *p*<00001. Scale bar = 160 μm

 The next set of experiments aimed to study the influence of the media flow as potent mechanobiological stimulus on the bone spheroids to further enchance the osteogenic environment. Consequently, we designed a fabricated microfluidic platform fitting into a miniaturized cultivation chamber (see also details in Fig. 1B). First, we tested the microfluidic system consisting of three individual channels with 60 microwells. Microwells with a diameter of 400 μm and a depth of 400 μm were situated in three lines with 100 μm space between each other, and the whole system was connected to the syringe pump (see also Fig. 1C). Cells at a concentration of 1 × 10^7^ cells per milliliter were introduced into the system by cell injection and placed into an incubator overnight. Then, the media flow was set to 2 µl/min, and the cells were cultivated for the next 3 days. The initial spheroids size generated in the closed microwell system was smaller (116 μm ± 23) than the spheroids formed in the open wells (154 μm ± 21) (Fig. 4G). Similarly like in the previous results, the spheroids decreased their mass during cultivation (Fig. 4G). We observed a decreased diameter of spheroids in the order from open microwells (99 μm ± 15), closed microwell platform (95 μm ± 14) to dynamic microwell platform (89 μm ± 21) after next 3 days of cultivation (Fig. 4A, B). The necrotic core formation of these spheroids was visualized by propidium iodide mainly in the centre of the spheroids (Fig. 4A´, B´) and did not show significant differences between dynamic and static conditions (Fig. 4H). As brief example to demonstrate the potential of murine bone spheroid model in regenerative medicine, fusion of spheroids to adult bone was investigated as shown in the supplementary materials (see Supp. Chap. 6). Tissue fusion is a crucial process in embryonic development and also plays an important role in the integration of tissue engineered constructs for regenerative strategies [7]. Also, a decrease of fusion capacity can be correlated with model immaturity [8]. Even though this experimental approach presents an interesting side aspect that highlights the fitness of our murine pre-osteoblasts, subsequent benchmarking activities of our optimized multi-plexed 3D spheroid systems focused on more basic analysis parameters that related to the bone physiological niche rather than regenerative capacity of spheroids.

Even though 3D microwell systems can be used potentially for a variety of applications in biomedical research, they evident lack of media perfusion is a limitation for many bone-on-chip studies, that require a highly dynamic tissue environment. Consequently, we designed and fabricated a micropillar platform forming a flow-through circular area to form bone models under physiologically more relevant conditions found in well-vascularized human bone. While pre-osteoblasts that were grown on flat micropillar chambers under comparable culture conditions as in the microwell system, this particular design that lacked microvacities did not promote any spheroid formation with cell growth on the entire biochip surface (Fig. 4C, D). Nonetheless, a positive effect of fluid flow on both viability (Fig. 4C´, D´) as well as mineralization staining analyzing Ca^2+^ intake was observable and comparable to that in the simple channel system (Fig. 4D), that lacked the more complex µpillar array. Next, we combined the microcavities as architectural cue for facilitated spheroid formation with µpillars that should simulate trabecular structures of bone tissue in a convergent approach shown in Fig. 4E, F. The depth of the microwells was designed to be only 30 μm to induce cell condensation but to present the effect of media force on 3D cell cultures, and the micropillar array prevents spheroids from being washed away from the surrounding area. The shallow microwells were sufficient for spheroid formation (Fig. 4E, F), but spheroids generated in these structures were smaller than spheroids formed in a closed microwell platform (i.e., 75 μm ± 15 versus 95 μm ± 14 as shown in Fig. 4G). On the other hand, the spheroids cultivated in the microwell-pillar structured dynamic chip showed decreased loss of spheroid mass during cultivation in contrast to static one, 75 μm ± 15 in static and 86 μm ± 18 in dynamic conditions (Fig. 4G), which was also evaluated by increased viability compared to that under static conditions (Fig. 4H). The ratio of live/dead cells in static and dynamic conditions was 1.9 (± 0.5) and 2.1 (± 0.6) for the microwell platform and 1.6 (± 0.3) and 4.2 (± 0.5) for micropillar chip respectively. These results are well correlated with the faster media exchange in microwell-pillar bone-on-a-chip platform than in static conditions or deep microwells (Supp. Chap. 6). Interestingly, a few spheroids that formed in the vicinity of the surrounding pillar structured readily fused and overgrew two pillars. This interaction strongly resembled the previous experiments on shperoid fusion (see Fig. 4E, F in comparison with Fig. 3A,B). Overall, we could established a combined microwell-micropillar system for multiplex bone spheroid formation and cultivation under dynamic conditions that represent a more suitable arrangement mimicking in vivo bone systems aiming and toxicology and drug testing with benefits of our platform approach being highlighted in Fig. 4I.

**Fig. 5:**
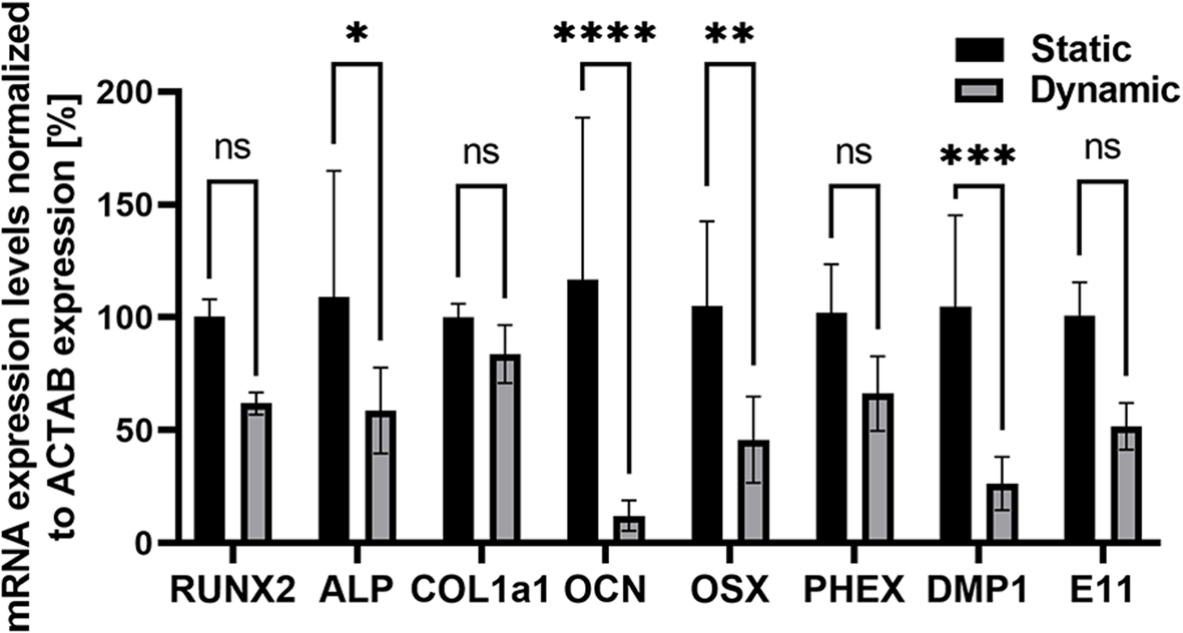
ACTAB-normalized mRNA expression levels of osteoprogenitor, preosteoblast, and osteoblast markers in the monolayer and spheroids were measured by qPCR. *n* ≥ 3, * *p *= 0.0459, ** *p* = 0.0078, *** *p* = 0.0003, **** *p *< 0.0001

To follow in more details the effect of the newly established platform design on osteogenic differentiation, we compared the mRNA expression of spheroids cultivated in the static and dynamic microwell-pillar platform. The analysis of the advanced cultivation routine was conducted under similar conditions throughout four days of cultivation comprising 1 day of static and 3 days of dynamic culture at a flow rate 2 µl/min. Surprisingly, most of the osteogenic differentiation markers were lower in dynamic conditions than static controls as shown in Fig. 5. The expressions were normalised to spheroids cultivated in static conditions. In detail, we observed expressiosn of RUNX2 (62 ± 5%, ns), ALP (59 ± 19%, *p* = 0.0459), COL1a1 (84 ± 13%, ns), OCN (12 ± 7, *p* < 0.0001), OSX (46 ± 19%, *p* = 0.0078), PHEX (66 ± 16, ns), DMP1 (26 ± 12, *** *p* = 0.003) and E11 (52 ± 10%, ns), which indicated the murine models sensitivity to fluid flow applications.

**Fig. 6:**
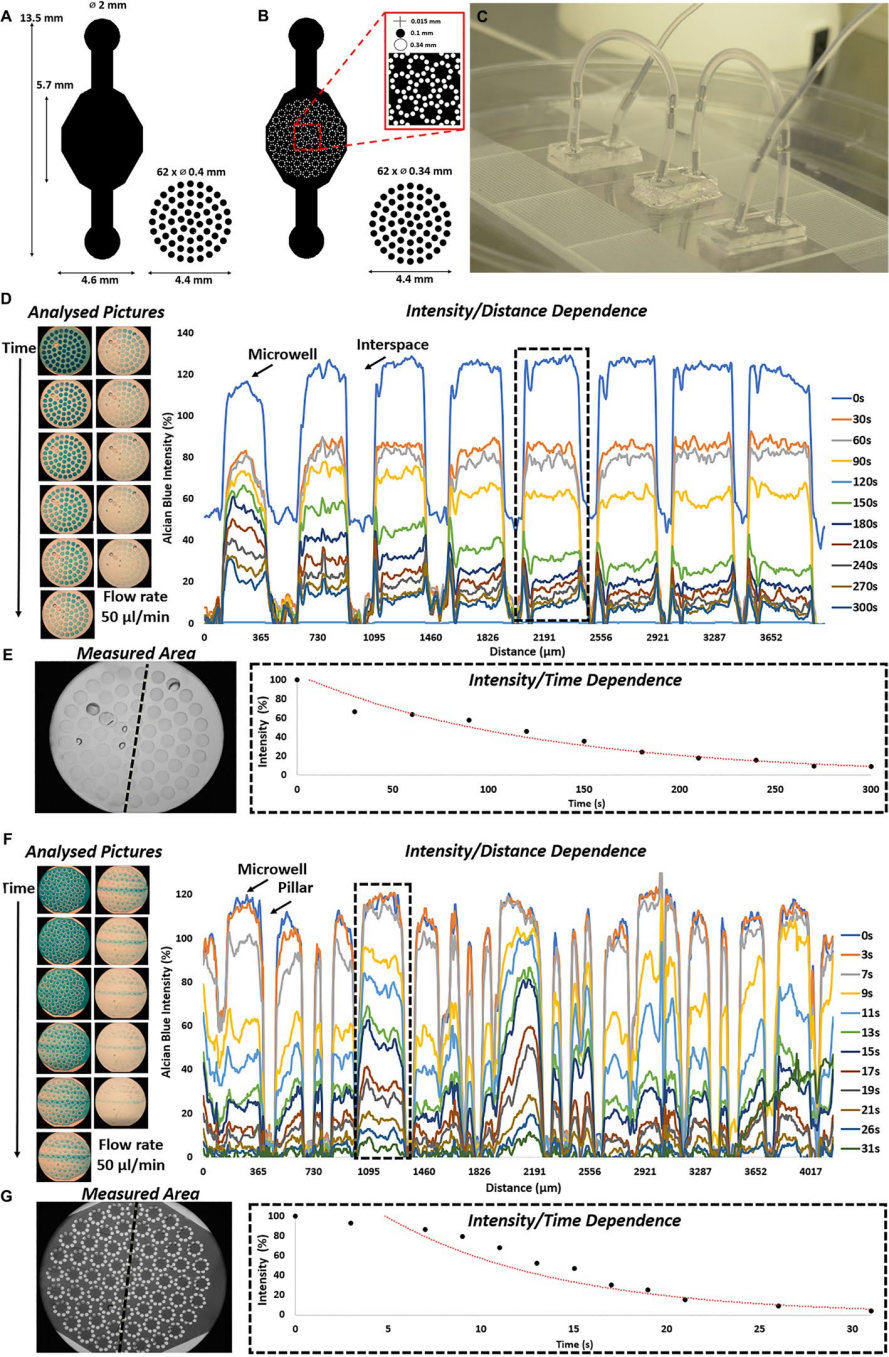
Schemes (**A** , **B**) and photographic image (**C**) of the mobile dynamic microfluidic culturing platforms suitable for cultivation inside an incubator connected to a linear setup (**C**). **D** , **F** Flow profile visualization and analysis of **E** microwell and **G** microwell-pillar bone-on-a-chip systems

To understand the apparent sensitivity of our model in more detail, we next used a less integrated mobile bone-on-a-chip platform capable of cultivate in a commercial incubator (Fig. 6A-C). We hypothesized that the attenuating effects of fluid flow on murine 3D biochip models were due to potential fluidic over-stimulation due to change of fluid distributions of the µpillar design. Due to the decrease of cross-sectional area by each and every pillar structure, the regional flow speed thus shear applied to the individual spheroids are being potentiated, which was tested in more detail for microcavity and micropillar chips. The residence time of a defined bolus of 1% Alcian blue solution, which was subsequently pushed through the culture region and finally washed out was investigated using ImageJ using a rather fast syringe pump set to 50 µl per minute. A complete washout cycle took approx. 300 s for the microwell design (Fig. 6D, E), and a 10-fold faster washout of the indicator dye of approx. 30 s in the microwell–pillar platform (Fig. 6F, G). This corresponds to calculated mean velocities of 15.3 and 153 μm/s and fluid shear of 0.005 dyn/cm^2^ and 0.05 dyn/cm^2^, respectively. These results suggest that the introduction of an array of µpillars can be used to elevate regional fluid shear to potentially modulate bone physiology aspects of spheroid models. We observed that fluid shear increase by 10-fold improves bone spheroid viability while it attenuated osteogenic potential of the murine bone 3D model. Overall, this can potentially point out at a certain oversensitivity of MCT3T3-E1-based murine spheroids for fluid actuation.

### Pilot study on the dynamic multiplex spheroid platform as unique opportunity for the study of human 3D bone models

**Fig. 7:**
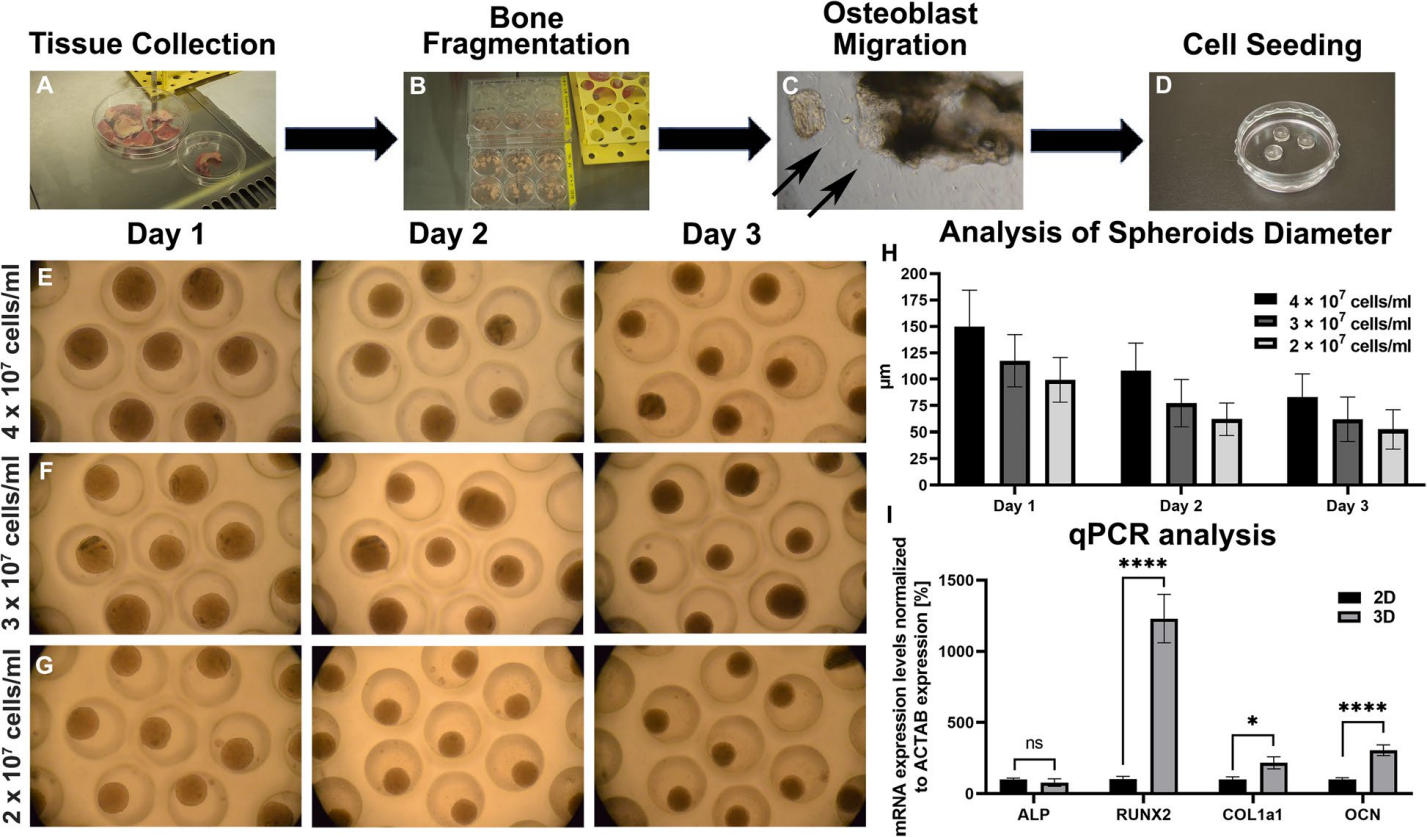
Generation of primary human bone spheroids. **A**-**D** Visualisation of the method for osteoblast isolation from human bone fragments. **E**-**G** Spheroid formation using the PDMS microwell platform cultivated for 3 days. **H** Analysis of spheroid diameters at different initial seeding densities during 3 days of cultivation (*n*  = 150). **I** Actin-normalized mRNA expression levels of osteogenic markers in the monolayer and spheroids were measured by qPCR. *n*  ≥ 3, * *p*  = 0.0145, **** *p*  < 0.0001. Microwell spheroids were captured under 100x magnification

As a final proof-of-concept, we prepared dynamic human bone spheroids-on-a-chip analyses again for microwell and microwell-pillar designs against 2D cultures to see whether the attenuating effects fluid flow on human-bone-derived spheroids derived from knee joint spongiosa were similar to the results of the murine MCT3T3-E1 spheroids, that were used for the optimization and characterization of the proposed microfluidic platform. To initially produce human baseline data on static bone models, we first established primary human osteoblasts from bones obtained after orthopaedic knee surgery (Fig. 7A) using a frequently used enzymatic isolation approach to generate human primary cultures of bone-derived cells (hBDCs). As shown in Fig. 7A-C, hBDCs readily migrated from bone fragments after collagenase II digestion (Fig. 7B, C, arrows) and after expansion, the cells were successfully seeded on the multiplexed spheroid microwell platforms (Fig. 7D). Even though we anticipated that human cells take longer to form spheroids due to donor age and differences in cell doubling capacity, we increased the initial concentrations and to our surprise the hBDC-derived spheroids were forming spheroids overnight (Fig. 7E-G). Notably, similar concentration dependent size decrease dynamics with regard to the previously analyzed murine counterparts were again observable. In brief, the re-optimized concentration of 4 × 10^7^ cells/ml led to the formation of the spheroids in the initial size of 149 ± 34 μm condensing to to 82 ± 22 μm after 3 days of cultivation (Fig. 7H). Reflecting on the condensation dynamics of the murine spheroids of initially 144 μm to 98 μm within a 3-day cultivation peroid, a 4-fold increase of hBDC seeding concentrations could effectively compensate for differences in cell growth and cell activity, and in turn produce spheroids of comparable size and maturation dynamics. For a more detailed look at specific bone mRNA markers, a reduced set of osteogenic markers were analyzed for human 3D bone spheroid cultures in comparison conventional 2D culture. As expected, not only the formation dynamics but also mRNA response patterns were improved similarly to the static murine spheroid models. Figure 7I shows that the mRNA expression levels of human-spongiosa tissue derived 3D spheroids displayed a significant increase in most of the bone markers including RUNX2 (1229 ± 170%, *p* < 0.0001), COL1a1 (216 ± 42%, *p* = 0.0145), and OCN (305 ± 37%, *p* < 0.0001).

**Fig. 8:**
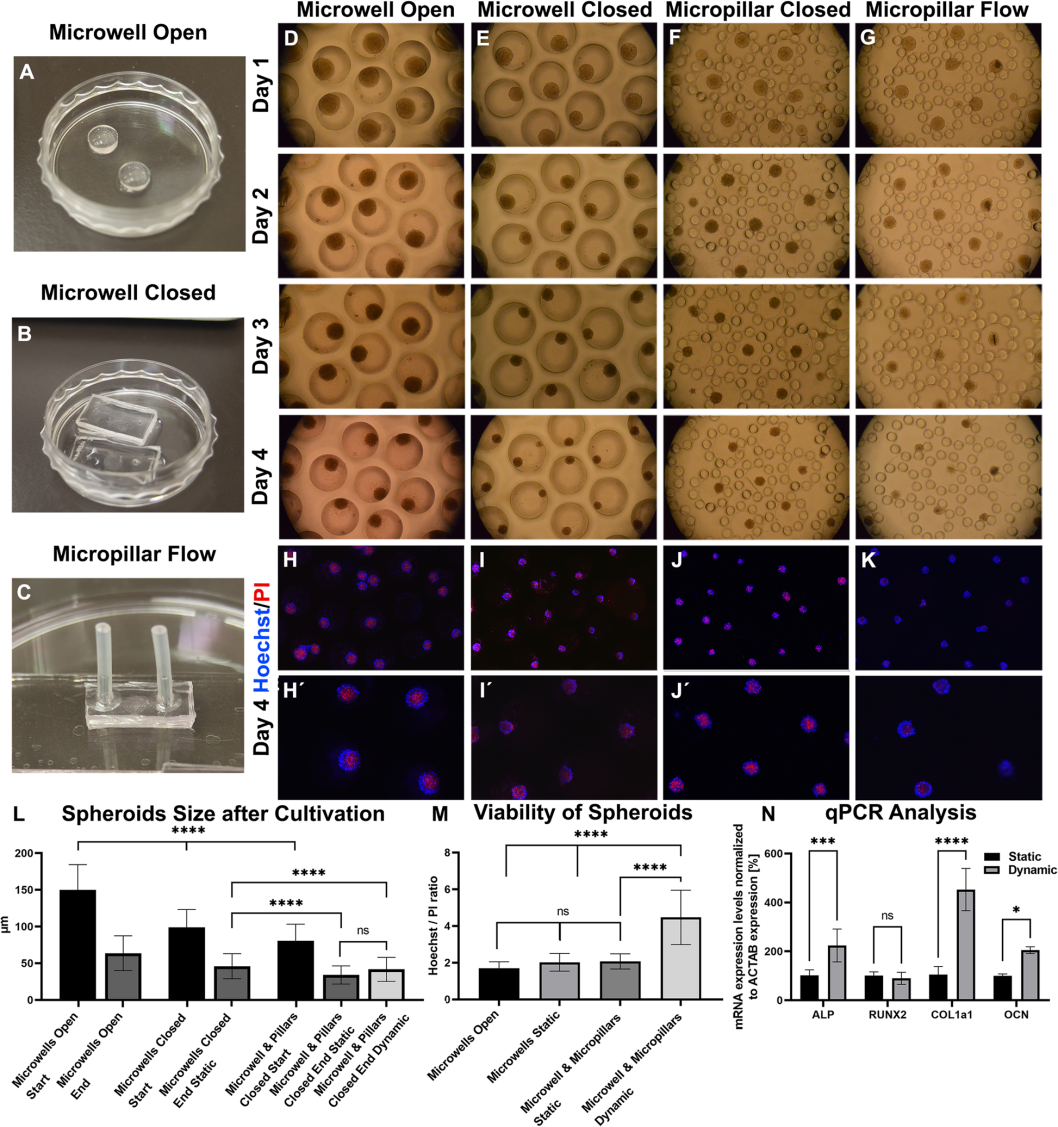
Comparison of microfluidic cultivation of human bone-cell-derived spheroids during a total of 4 days of cultivation. Spheroids cultivated in the open microwells (**A**) exhibited significantly higher size compared to closed platforms (**B**, **C**, **D**). **L** Graphical analysis of spheroid mass. The closed microwells (**B**) produced significantly higher spheroids than static closed microwell-pillar platforms (**C**). There was no significant difference between the dynamic microwell-pillar platform (**D**) and the closed microwells (**B**). **E**-**H** Propidium iodide staining showed the positive effect of dynamic cultivation on spheroids viability (**M**). **I**, **J**, **K** Photos of the used platforms. **J** Relative mRNA expression levels of osteogenic markers of pooled spheroids using the 3D cell culture platform after 3 days of cultivation under dynamic conditions in comparison to static on-chip cultivation regimes. **L** *n* = 120, **** *p* < 0.0001. **M** *n* = 70, **** *p* < 0.0001. **J** Data are expressed as mean ± SD relative to beta-actin housekeeping gene for *n* ≥ 2 (Two-way ANOVA with Tukey’s post-hoc test *** *p* = 0.0002, **** *p* < 0.0001). Platforms for spheroid mass measurement were captured under 100x magnification. PI staining was analysed for 60x and 100x magnification objectives

As a final set of experiments, the response of the primary human bone model to a more dynamic bone-tissue-like cultivation environment was investigated using gradually increasing complexities from open microwells up to fully perfused bone-on-a-chip platforms (Fig. 8A) including static approaches such as the open microwell platform, closed microwell and microwell-pillar designs, as well as more dynamic conditions tested with the microwell-pillar platform with tubes (Fig. 8D) at a flow rate of 2 µl/min through day 4. In brief, the hBDC-derived spheroids for a optimized initial seeding density showed comparable results to the murine models with a decrease of the spheroid size to around 70–100 μm (Fig. 8L) while again improving the viability of the spheroidal bone construct when activating the more physiological dynamic flow conditions. While human spheroids cultivated in the open platforms were the highest in size with a diameter of 63 ± 23 μm after 3 days (Fig. 8D, L), static closed microwell (45 ± 17 μm; Fig. 8E, L) and microwell-pillar platforms (34 ± 12 μm; Fig. 8F, L) showed a slight decrease of spheroid diameters independent of dynamic conditions while significantly improving spheroid viability expressed by calcein/PI ratio by more than 2-fold of the open microwells. Overall, these re-characterization results for the hBDC derived bone spheroid models indicate that human primary cells show higher sensitivity for the static cultivation approaches but retain an improved performance when applying dynamic protocols using fluid perfusion in combination with micropillars.

As a final effort to demonstrate the successful development of the microfluidic platform technology for not only murine but more importantly human bone models, the gene expression response to the dynamic cultivation protocol again was compared to static biochip cultivation routines to investigate potentially beneficial effects of the media flow on osteogenic markers including ALP, RUNX2, COL1a1 and OCN in the proposed advanced spheroid array platform (Fig. 8J). Notably, the level of ALP (224 ± 67%, *p* = 0.0002) and COL1a1 (452 ± 86%, *p* < 0.0001), OCN (204 ± 14%, *p* = 0.0043) was significantly higher in dynamic conditions than in static ones. On the other hand, the dynamic cultivation did not affect the expression of RUNX2.

## Discussion

In this project, we successfully designed and fabricated a miniaturized microsystems platform to investigate murine and human 3D bone models inside microfluidic channels under more dynamic culture conditions while retaining the online monitoring capabilities of optical microscopy approaches. We iterated and characterized several and gradually increasing microfluidic chip designs to finally approximate the chip capabilities by additional chamber structuring by µpillars to resemble trabecular bone tissue characteristics for advanced and dynamic 3D bone cell cultivation.

Most primitive 2D murine preosteoblast MC3T3-E1 investigations under dynamic conditions triggering increased levels of Ca^2+^ intake and a change in cytoskeleton rearrangement were in line with the fluid mechanical responsiveness of bone cells by fluid flow [9]. Notably, the increase in the extracellular entry of Ca^2+,^ which is important for ERK1/2 signaling regulation in osteoblast mineralization [10, 11] and our findings confirm previous reports on dynamic MC3T3-E1 experiments [12, 13]. with rearrangement of the F-actin stress fibres and increase of osteogenic markers including OCN, COL1, and RUNX2. Such basic fluid dynamic responses we previously confirmed also using different bone-derived cell types [14–18] including also human progenitor dermal fibroblasts and the embryonic stem cell-derived mesenchymal progenitor cell line cultured under dynamic conditions [19]. We also demonstrated that multiplexed murine bone spheroids are a promising in vitro screening tool to study osteoblast differentiation capacity analyzed by i.e. the expression of RUNX2, ALP, PHEX, OCN, OSX, DMP1 and E11. RUNX2 is a transcription factor that induces the commitment of progenitors to the osteoblast lineage and controls the proliferation of preostoblasts as well as the expression of ECM proteins [20]. ALP is involved in ECM mineralization and enables the formation of hydroxyapatite during the transformation of osteoids into mineralized ECM essential for the differentiation of osteoblasts towards osteocytes [21]. PHEX expression is then described in osteocytes and is also associated with ECM mineralization [22], similar to OCN [23]. OCN expression is induced by the transcription factor OSX, which is an essential factor for osteoblast differentiation and ECM mineralization [24]. Significantly increased expression of DMP1 (involved in mineralization) and E11 (role in dendrite elongation) indicates successful induction of late osteoblast differentiation in the 3D system [25, 26]. Taken together, our results confirm that 3D models outperform 2D bone cell cultures with regard to both osteogenic differentiation capacity as well as vital gene regulation necessary for the initiation of the transformation of the osteoblast into its terminally differentiated osteocyte similar to previous works on static models [1–3].

Modern microfluidic methods represent unique opportunities to approximate towards a very complex in vivo bone environment thus providing the missing link between too simple in vitro models and very time consuming in vivo model systems (i.e. bone marrow models [27] or test animals). While most microfluidic 3D bone platforms are mainly based on the implementation of the organic and inorganic scaffolds such as titanium fibre mesh scaffolds [28], porous polymer scaffolds [29], polyurethane scaffolds [4], poly(lactide-co-glycolide) scaffolds [30] and even more natural collagenous matrix [31], in the current study we established a novel convergent approach that combines the inherent capabilities of primary cells to form bone tissue-like spheroids with a dense array of µpillars inside the microfluidic channels to form a fluid mechanical osteogenic interface that resembles the architecture of trabecular bone. In spite of many studies covering the dynamic response of bone cells, dynamic scaffold-free 3D bone platforms are still very rare [32]. Spheroids are the most promising technology to speed up pharmaceutical drug development and high throughput screening applications. The technological complexity thus huge intrinsic bias/challenge to develop bioengineered systems for the complex task of emulating bone biology is not surprising as it requires transdisciplinary research that requires extensive engineering, biological as well as medical expertise. To overcome some arising challenges and issues, we here developed a stand-alone bone-on-a-chip platform to show that a detailed characterization with development of robust cultivation routines and analysis protocols can speed up and facilitate this biomedical hurdle. Here, for the first time and to the best of our current knowledge, we report a microwell-micropillar platform for establishing and cultivating 3D cultures under more relevant fluid dynamic conditions while also emulating bone tissue like architectures with integration of µpillar arrays. In line with pioneer work from the cleanroom to introduce pillars to model trabecular bone [33] which was also tested under static conditions [34]. Although the pillar surface alone triggered cellular sphere formation similar to biomimetic nanostructures, the novel combination of microfluidic microwells crucial for reproducible spheroid formation and a µpillar that not only prevent washing out of spheroids but also emulates trabelular structures within a dynamic bone tissue-like microenvironment represents a novel tool for studying a variety of patho/physiological, pharmaceutical and also toxicological processes using advanced and more functional, dynamic microsystems.

In a pilot study on human bone-derived primary cells, we achieved to confirm previous studies on the beneficial effect of a 3D tissue-like context compared to conventional 2D primary osteoblasts with upregulation of e.g. osteogenic markers such as osteopontin and collagen type I [35]. Conventional bone tissue engineering approaches using bioreactors set out to trigger immunobiological expresion (e.g., OPN, OSX) for periods like 30 days of cultivation [36] and more [37]; however, these strategies often cannot produce enough samples sizes to be attractive for state-of-the-art screening appliations. In the current study, we specifically aimed to establish a rapid and reproducible multi-plexed screening approach with optimized protocols to not only form murine but also human bone spheroids within 3 days, and to create an integrated technology platform that can further introduce microsystems concepts in future interations to provide more (i) automation steps (i.e., microvalving and µpump integrations), (ii) scale-up by state-of-the-art production processes (i.e., relica molding and potentially also embossing and injection molding), and (iii) further integrated with modern optical imaging capabilities (i.e., multi-photon imaging and AI-based data analysis). Another vital aspect that we wanted to demonstrate is how a well-integrated biomimetic spheroid-based microsystemic approach can lead to a rapid time-to-result duration (i.e., three days from seeding to endpoint) because it can significantly improve an pro-osteogenic environment needed to experiments on potential known stimulative and inhibitory agents, as well as the next generation of bioactive molecules and therapeutic startegies aiming to modulate bone patho/physiology.

## Conclusions

Here, we report a microfluidic-based platform for studying osteoblast physiology using 3D cell cultures. Moreover, we developed a small incubation unit for online monitoring. The microwell platform can serve as a powerful tool for drug testing and research, but it is sufficient for fluid stress-inducible cell cultures. For the first time, we also introduced a microwell-micropillar system that enables spheroid formation and cultivation in a dynamic fluidic environment. Our results indicate that primary human osteoblasts can be used for studying osteoblast patho/physiology in multiplexed 3D cell spheroid cultures. Moreover, the developed platform technology can be easily connected to a media flow system to mimic the dynamic environment of trabelular bone tissue. We envision our advanced microsystems approach to human bone modelling to improve biomedical applications such as toxicology, drug testing as well as disease modelling such as osteosarcoma or osteoporosis. We believe that our microwell-pillar platforms represents a unique opportunity as complimentary technology to existing bone models that can be potentially not only used in classical applications but may even find a place in speeding up personalized drug testing or even research in regenerative medicine in murine models [1, 38, 39] and some day even human patients [40, 41].

## Materials and methods

### The design and fabrication of a miniaturized optically accessible cultivation chamber

The design of the microdevice was modelled in computer-aided design software (Fusion360, Autodesk Inc.) and manufactured from plexiglass by a milling machine with computer numerical control. We set out to design a miniaturized chamber for online cultivation monitoring of cells under standard conditions, i.e., 5% concentration of CO_2_ and 37 °C. The device consists of a chamber body fabricated from optical transparent plexiglass of the shape of a medium Petri dish (inner area of 22.1 cm^2^) to be compatible with a standard inverted microscope table. The chamber with a diameter of 60 mm and height of 16 mm consists of the upper cover part with ports for CO_2_ influx (φ 4 mm), a CO_2_ detector (COZIR-WX-20, AirTest, Canada) and a resistor thermometer with a pt100 sensor (φ 1 mm) and the lower part body with 6 grooves for tightening the capillaries prepared from needles and port for the pt100 sensor. The chamber body also contains a circular groove with a width of 5 mm and a height of 5 mm to hold approx. 4 ml of the water around the cultivation room for internal humidification. The chamber also contains an anchoring ring from high-strength aluminium and four plastic screws that hold various microfluidic inserts inside the body of the chamber. For the detailed design (Supp. Chap. 1) of our chaber and whole system connection (Supp. Chap. 2, 4), see the Supplement.

### Monitoring and controlling microfluidic operations

The content of CO_2_ and temperature inside the chamber were precisely regulated by external sources to obtain an autonomous system for online monitoring and cultivation inside the incubator placed at the microscope table. The temperature system is composed of a silicon heating belt (192 × 10 mm, power 15 W Malapa, CZ) attached to the sidewall around the cover of the chamber by a fabricated Teflon ring powered by a thermoregulator (Omega, USA). The Teflon ring with a heating belt is connected to the cover to avoid water condensation inside the CO_2_ detector. The thermosensor (pt100) is positioned inside the body of the chamber as close as possible to the cultivation polydimethylsiloxane (PDMS) insert to take the temperature reliably. CO_2_ was regulated using PLC (Simatic S1200 1212 C DC/DC/DC, Siemens) programmed using Tia Portal (Siemens) and connected to the regulation system containing a CO_2_ bomb with a reducing valve, filter (5 μm, max pressure 1 MPa), subfilter (0.01 μm), electromagnetically operated 2/2 process valve for air, and throttle valve with a nonreturn valve. The signal from the regulator was transmitted to a computer and visualized using GasLab software (see Supp. Chap. 2). The media flow was controlled using syringe pumps (SyringeONE, NewEra, USA), and the whole system was connected to an inverted microscope Olympus IX 71 (Japan).

### Mold fabrication

The microfluidic inserts for cultivation were fabricated from one PDMS layer bonded to a glass slide or two bonded PDMS layers. Each layer was designed in CleWin5 software (WieWeb Software) and developed using molds using the direct writing photolithography system MicroWriter ML3 Pro (Durham MagnetoOptics, UK) and SU-8 polymer (MicroChem, USA). For a detailed fabrication process, see Supp. Chap. 3.

### Microfluidic inserts

The molds were covered by PDMS Sylgard 184 (Dow Corning, MI, USA) mixed from the base and curing agent at a ratio of 10:1 and degassed under vacuum for 20 min. The molds with PDMS were cured on a hot plate for 2 h at 80 °C. The PDMS slab was cut with a razor and bonded to glass or another PDMS layer after treating both surfaces with oxygen plasma, PDMS-glass for 60 s and PDMS-PDMS for 45 s.

In the case of a simple microchannel platform, the upper microchannel part was bonded to microscopic glass, forming three 200 μm high channels. In the microchannel-microwell PDMS platform, both layers were treated with oxygen plasma and bonded together, forming three independent 200 μm high microchannels for media flow and sixty 400 μm deep microwells in each channel for 3D cell culture formation and cultivation. The micropillar-microwell platform was then fabricated using two bonded layers of PDMS composed of an upper 200 μm high microchannel with a 200 μm high micropillar with a diameter of 100 μm and 15 μm spaces to create sixty cultivation rooms for cell condensation with a diameter of 360 μm. The bottom part then consists of a total of sixty microwells in each channel with a diameter and depth of 340 μm and 30 μm corresponding to pillar-formed cultivation rooms.

The PDMS microfluidic platforms were then individually placed into a miniaturized chamber, and the whole cultivation assembly was fixed by screws. The interface between the PDMS microfluidics and the chamber was formed by a layer of PDMS above the cultivation room (flow part of microfluidics), providing the full diffusion of CO_2_ into the cultivation space.

Except for simple channel experiments, all platforms were coated by a nonadhesive agent (Pluronic F-127 1:200, Thermo Fisher, USA) to prevent cell adhesion.

### Cell culture

A mouse osteoblastic precursor cell line, MC3T3-E1, was obtained from the European Collection of Cell Culture (c.n. 99,072,810). Proliferation conditions were prepared by the cultivation of the cells in MEM Alpha medium (αMEM; Gibco, USA) enriched by fetal bovine serum (10%, Gibco, USA) and penicillin/streptomycin (100 U/ml, 100 µg/ml). The differentiation conditions were adjusted by cultivating the cells in a differentiation medium prepared as described above but with the addition of 10 mM β-glycerolphosphate and 50 µg/ml ascorbic acid.

Human primary osteoblasts were obtained from female patients (*n* = 3) at the age from 68 to 81 with gonarthrosis undergoing knee replacement surgery. The fragments were separated from soft tissues and rinsed with sterile PBS with 1% P/S several times to wash out red blood cells. From the well-formed tissue without any pathological appearance, trabecular and cortical bone was separated from the rest of the tissue by bone rongeur and fragmented into small pieces. Fragments were transferred into tubes with PBS with 1% P/S and vortex. The supernatant was dismissed and the whole procedure was repeated three times. Then the 0.25% Trypsin was added and incubated at 37 °C in the shaker for 10 min. After the incubation the PBS with 1% P/S was added and the rinse of the fragments by PBS was repeated three times. After rinsing steps, the PBS was removed, Collagenase II (1 mg/ml or 300U/ml, Sigma‒Aldrich, USA) was added, and the fragments were incubated at 37 °C in the shaker for 30 min. Then the PBS with P/S was added, and fragments were rinsed by vortexing and changing the PBS solution three times. Then the PBS was removed, and fresh media was added. The fragments were vortexed last time, and the media was exchanged for fresh ones. The fragments were seeded to 6 well plates for initial osteoblast migration and media was changed every 4 days. The bone fragments were carried out after enough cells around individual fragments. After the 90% confluency (2–4 weeks), the osteoblast was transferred to the cultivation flask. Proliferation conditions were prepared by the cultivation of the cells in MEM Alpha medium (αMEM; Gibco, USA) enriched by fetal bovine serum (10%, Gibco, USA) and penicillin/streptomycin (100 U/ml, 100 µg/ml). The differentiation conditions were adjusted by cultivating the cells in a differentiation medium prepared as described above but with the addition of 10 mM β-glycerolphosphate and 50 µg/ml ascorbic acid.

### Fluorescence microscopy

For online monitoring of live cells, Calcein AM (1:1000, Invitrogen, ThermoFisher, USA), propidium iodide (1:1000, ImmunoChemistry Technologies, USA) and Hoechst 33,342 (1:1000, ImmunoChemistry Technologies, USA) were used for nuclei visualization.

For cytoskeleton visualization, cells were fixed with 4% paraformaldehyde for 20 min and washed with PBS. Cells were stained with ActinGreen 488 ReadyProbe (Invitrogen, ThermoFisher, USA) for actin and with Hoechst 33,342 for nuclear staining. Then, the cells were stained with 2% alizarin red for 20 min to evaluate mineralization. Samples were observed with an inverted microscope Olympus IX 71 (Japan) and merged in Adobe Photoshop (USA).

### RNA isolation and molecular analysis

For 2D and 3D comparison, we used at least three PDMS platforms for one sample (approx. 180 spheroids). The platforms were transferred to 2 ml by tweezers and vortexed in media to wash out the pheroids from wells. Then the platforms were collected and tubes with spheroids were quickly centrifuged. The media was removed, and the spheroids were lysed in 100 µl of RLT lysis buffer (Qiagen) with β-ME (Sigma‒Aldrich, USA). The spheroids for static and dynamic 3D comparison were lysed inside the microfluidic chips. The 100 µl of RLT lysis buffer with β-ME was added for each platform. One sample composed of 2 individual platforms/channel, thus total 200 µl of lysis buffer. Total murine RNA was extracted from cells using the RNeasy Mini Kit (Qiagen, Germany) and innuPREP RNA Mini Kit (innnuCREEN, Germany) for human samples. cDNA was synthesized using the gb Elite Reverse Transcription Kit (Generi Biotech, Czech Republic). The TaqMan Gene Expression Assay (Thermo Fisher Scientific) was applied to detect the gene expression of RUNX family transcription factor 2 (RUNX2 Mm00501584_m1), alkaline phosphatase (ALP, Mm00475834_m1), collagen type I alpha 1 chain (COL1a1, Mm00801666_g1), osteocalcin (OCN, Mm03413826_mH), osterix (OSX, Mm00504574_m1), phosphate regulating endopeptidase X-linked (PHEX, Mm00448119_m1), dentin matrix acidic phosphoprotein 1 (DMP1, Mm01208363_m1) and E11/podoplanin (E11, Mm00494716_m1). The expression levels were calculated using the ΔΔCT method with normalization based on actin levels (Actb, Mm02619580_g1).

The human samples were lysated by 350 µl RLT lysis buffer with β-ME, the reverse transcription was done by High-Capacity cDNA Reverse Transcription Kit (Applied Biosystems, USA). The tags for qPCR reaction were prepared by (Metabiom, GE) with the specific sequences for primers (ACTAB: f: CTGGAACGGTGAAGGTGAAC, r: AAGGGACTTCCTGTAACAATGCA; RUNX2: f: TGCGGTGCAAACTTTCTCCA, r: GCTTGCAGCCTTAAATGACTCTG; ALPL: f: TGATGTGGAGTATGAGAGTGAC, r: TGAAGTGGGAGTGCTTGTATC; BGLAP, f: GCAGAGTCCAGCAAAGGTG, r: CCAGCCATTGATACAGGTAGC; COL1a1, f: CACTGGTGATGCTGGTCCTG, r: CGAGGTCACGGTCACGAAC). The expression levels were calculated using the ΔΔCT method with normalization based on actin levels.

### Statistics

Results were calculated with GraphPad Prism. Simple columns statistics was performed using Tukey’s multiple comparisons test. Multicolumms samples that were not normally distributed were analysed using one-way ANOVA or two-way ANOVA test followed by Tukey’s multiple comparison test.

## Supplementary Information

**Additional file 1.**

## Data Availability

The datasets used and/or analysed during the current study are available from the corresponding author on reasonable request.
