# Correction: Microfluidic device for enhancement and analysis of osteoblast differentiation in three-dimensional cell cultures

**DOI:** 10.1186/s13036-025-00501-3

**Published:** 2025-04-16

**Authors:** Michael Killinger, Adéla Kratochvilová, Eva Ingeborg Reihs, Eva Matalová, Karel Klepárník, Mario Rothbauer

**Affiliations:** 1 Department of Bioanalytical Instrumentation, Institute of Analytical Chemistry, Academy of Sciences, Brno, Czech Republic; 2 Department of Chemistry, Faculty of Science, Masaryk University, Brno, Czech Republic; 3 Laboratory of Odontogenesis and Osteogenesis, Institute of Animal Physiology and Genetics, Academy of Sciences, Brno, Czech Republic; 4 Cell Chip Group, Institute of Applied Synthetic Chemistry, Institute of Chemical Technologies and Analytics, Faculty of Technical Chemistry, Technical University Vienna, Vienna, Austria; 5 Karl Chiari Lab for Orthopaedic Biology, Department of Orthopedics and Trauma Surgery, Medical University of Vienna, Vienna, Austria

**Correction** **:** **J Biol Eng 17, 77 (2023)**

**https://doi.org/10.1186/s13036-023–00395-z**

Following the publication of the original article [1] we were informed that an old version of Figure 4 was accidentally provided during the manuscript's submission.

In particular, panel 4G of the Figure showed remnants of data that was revised prior to the manuscript's acceptance for publication.


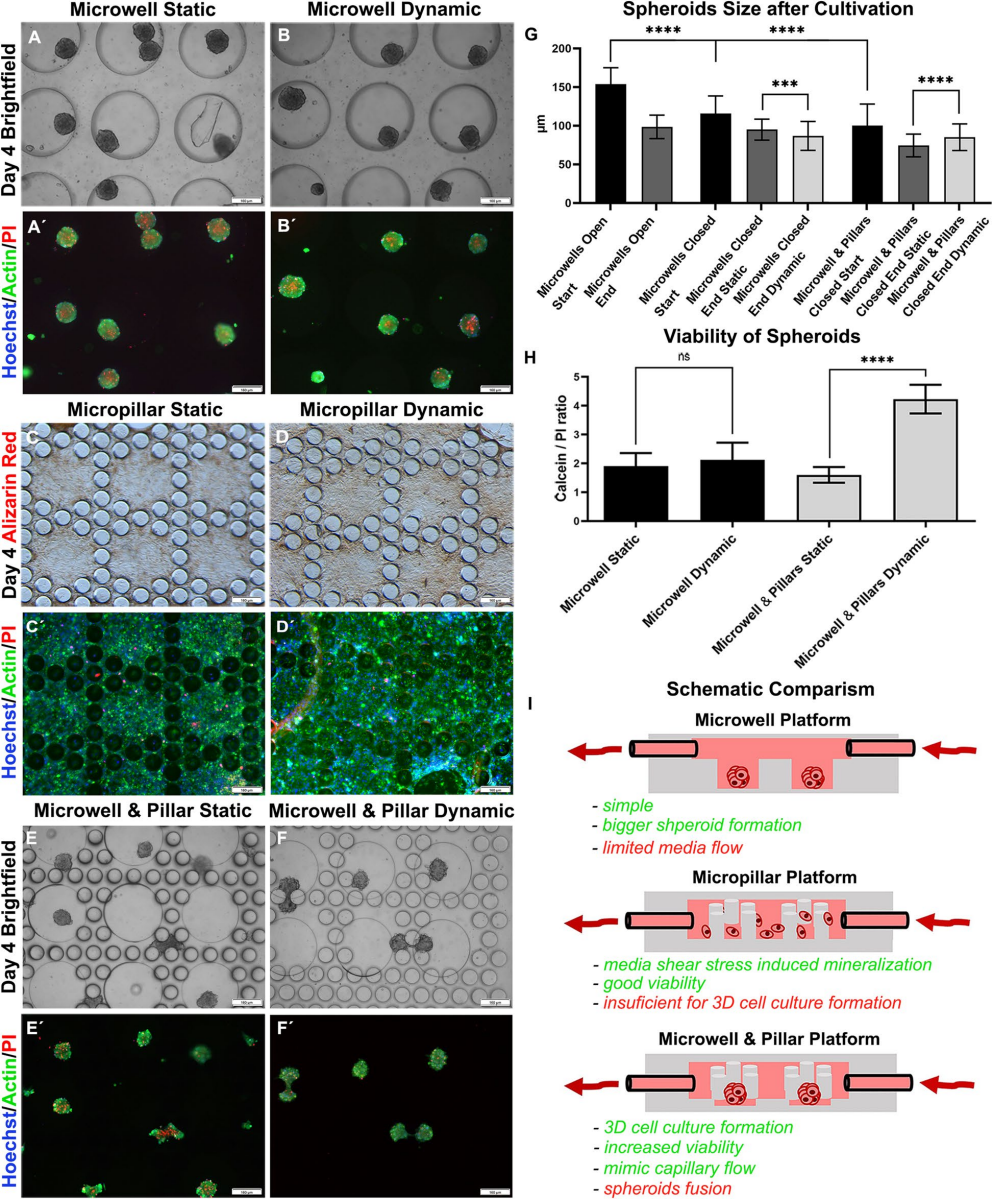


The correct Figure 4 is shown in this Correction. The original article has been corrected.
