# Supplementary material for: Microfluidic device for enhancement and analysis of osteoblast differentiation in three-dimensional cell cultures
Source: J Biol Eng. 2023 Dec 14;17:77. doi: 10.1186/s13036-023-00395-z (PMC10722696; doi:10.1186/s13036-023-00395-z)

Supplementary Information

-

Microfluidic Device for Enhancement and Analysis of Osteoblast Differentiation in Three-Dimensional Cell Cultures

Michael Killinger[1,2], Adéla Kratochvilová[3], Eva Ingeborg Reihs[4,5], Eva Matalová[3], Karel Klepárník[1+], Mario Rothbauer[4,5*+]

[1]Department of Bioanalytical Instrumentation, Institute of Analytical Chemistry, Academy of Sciences, Brno, Czech Republic

[2]Department of Chemistry, Faculty of Science, Masaryk University, Brno, Czech Republic

[3]Laboratory of Odontogenesis and Osteogenesis, Institute of Animal Physiology and Genetics, Academy of Sciences, Brno, Czech Republic

[4] Cell Chip Group, Institute of Applied Synthetic Chemistry and Institute of Chemical Technologies and Analytics, Technical University Vienna, Faculty of Technical Chemistry, Vienna, Austria

[5] Karl Chiari Lab for Orthopedic Biology, Department of Orthopedics and Trauma Surgery, Medical University of Vienna, Vienna, Austria.

[+] *Co-last authorship*

*** Correspondence:** Mario Rothbauer, mario.rothbauer@tuwien.ac.at, mario.rothbauer@meduniwien.ac.at

Keywords: bone-on-a-chip, 3D cell cultures, dynamic cultivation, microfluidics, microwells micropillars

Content:

1. **Miniaturized Incubator - Design**
2. **Miniaturized Incubator – Whole Assembly**
3. **Glass Molds Fabrication**
4. **Tubing**
5. **Mobile Platforms Fabrication**
6. **3D Cultures in Bone Regeneration**

1. Miniaturized Incubator - Design

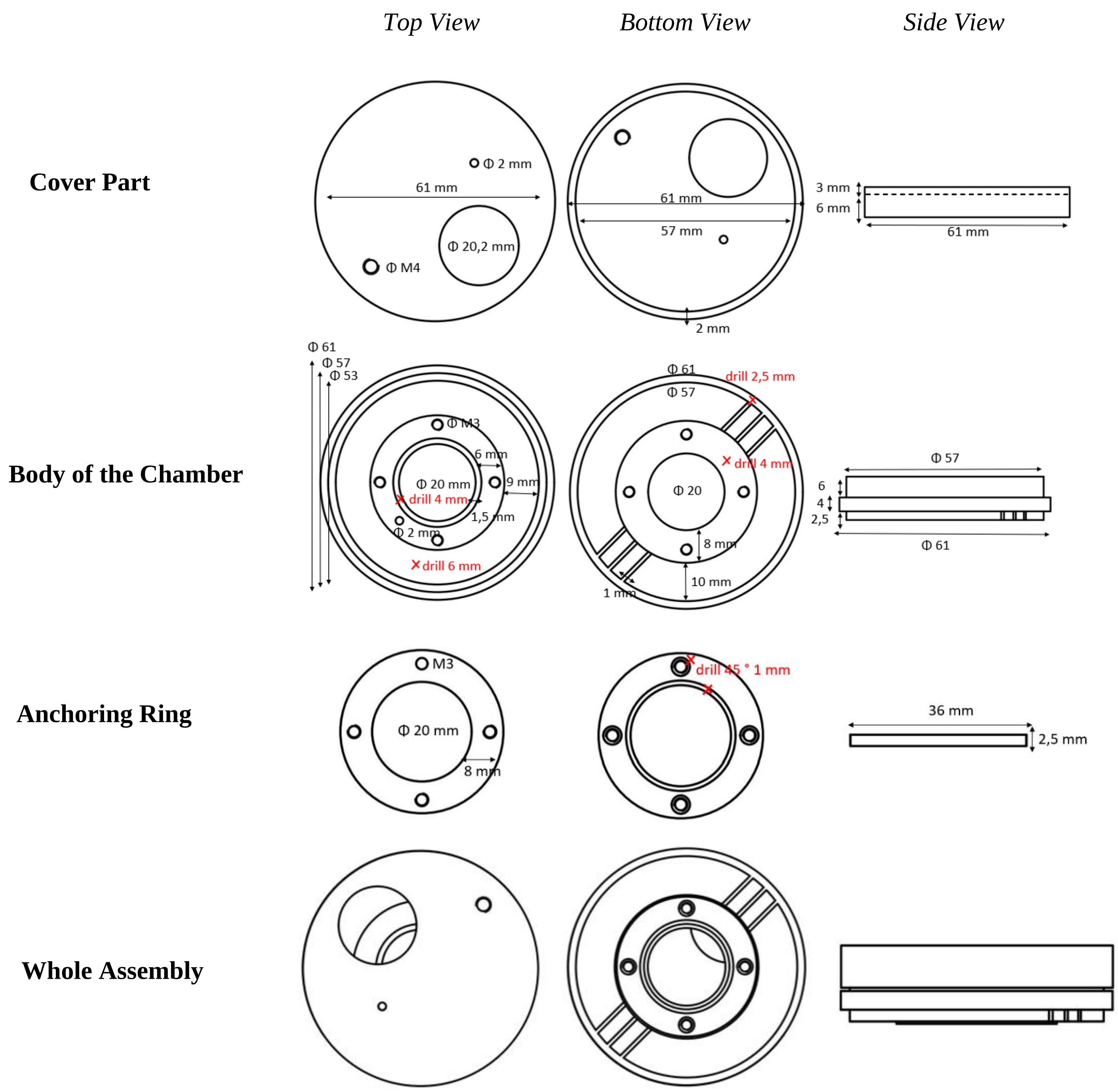

2D sketch of the miniaturized cultivation chamber and incubator for parallelized cultivation. The system consists of a cover and body in the size of a medium Petri dish and an anchoring ring for tightening PDMS microfluidic by screws.

2. *Miniaturized Incubator – Whole Assembly*

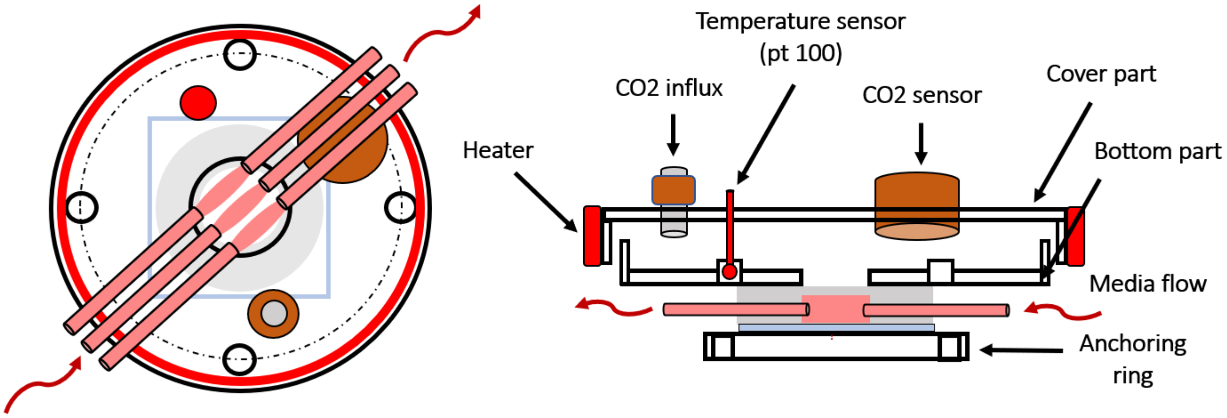

Graphical scheme of the top view and transversal section of the cultivation chamber. The chamber consists of an independent CO_2 supply and detection (brown). The heat detector (pt100) is situated near cultivation. The heating ring is placed on the bottom part of the cover to eliminate the humidity around the CO_2 detector that influences CO_2 detection.

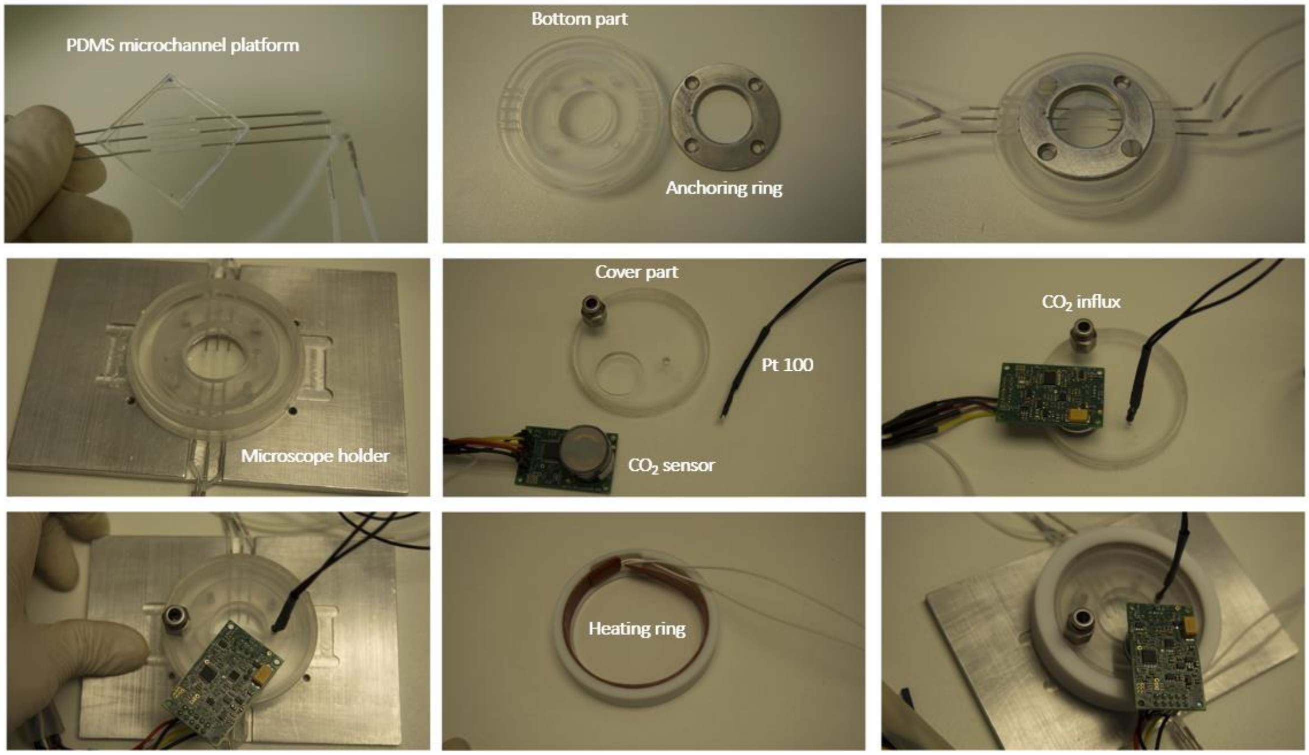

The whole assembly and preparation of the cultivation chamber. The PDMS layer containing holes and capillaries for medium influx and efflux is placed on the lower part of the cultivation dish and covered by a glass plate (not in the case of the bonded PDMS-PDMS platform) for spheroid cultivation. The glass plate is then covered by a stainless-steel plate screwed to the chamber to prevent medium loss. The body of the chamber is

plated on a microscope holder. The cover part of the chamber contains the positions for the CO_2 detector and influx tube and hole for the thermosensor (pt100). The prepared chamber is closed by the chamber cover. The electrical heater is then placed around the cover using a Teflon ring. The whole microfluidic system is then plated on the microscope table.

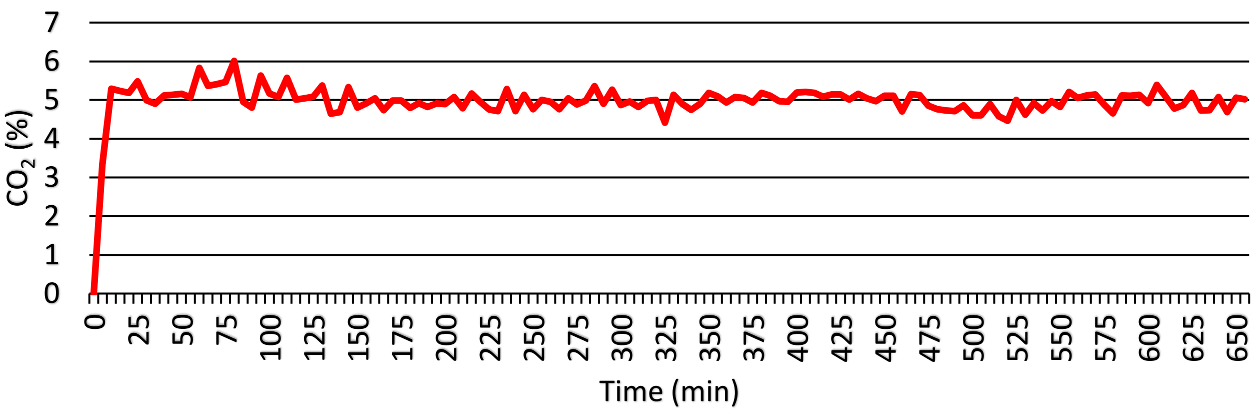

Stability of CO_2 concentration inside the cultivation chamber.

3. *SU-8 Molds Fabrication*

A simple mold for the microchannel platform was fabricated from a clean 5×5 cm Borofloat glass wafer coated with one 200 μm layer of SU-8 2100 (MicroChem, USA). Spin-coating was performed using a spin coater (Laurell WS 400B 6NPP LITE, USA) for 10 s at 500 rpm and 30 s at 1500 rpm. After spin coating, the glass was baked at 65 °C for 5 min and 95 °C for 60 min and exposed to ultraviolet (UV) light (500 mJ/cm2) in a direct photolithograph. Mold was developed in mr-Dev 600 (Microresist, Germany) after a short postbake period (65 °C for 5 min, 95 °C for 15 min).

The microchannel-microwell mold is composed of an upper-media flow mold formed the same as a simple microchannel mold and a bottom-micropillar part fabricated from a wafer coated with two layers of SU-8 2100 to obtain a depth of 400 μm (spin coating 10 s, 500 rpm and 30 s 1500 rpm followed by baking at 65 °C for 5 min and 95 °C for 60 min). The baking step in the case of the second layer was increased to 95 °C for 120 min. The coated glass was exposed to UV light (900 mJ/cm2) and developed in mr-Dev 600 after a short postbake period (65 °C for 20 min).

Finally, a micropillar microwell platform was fabricated from an upper-media flow mold (micropillar part) formed the same as a simple microchannel mold but with sixty 100 μm wide wells forming a circle with a diameter of 360 μm and 15 μm spaces between individual wells. The bottom part (microwell part) was made from 30 μm high structures with a diameter of 340 μm. The mold was developed from SU-8 3025 (MicroChem) using spin coating for 10 s at 500 rpm and 30 s at 3000 rpm postcoating baking at 65 °C for 5 min and 95 °C for 15 min. After exposure to 350 mJ/cm2, the mold was postbaked at 65 °C for 5 min and 95 °C for 5 min followed by development.

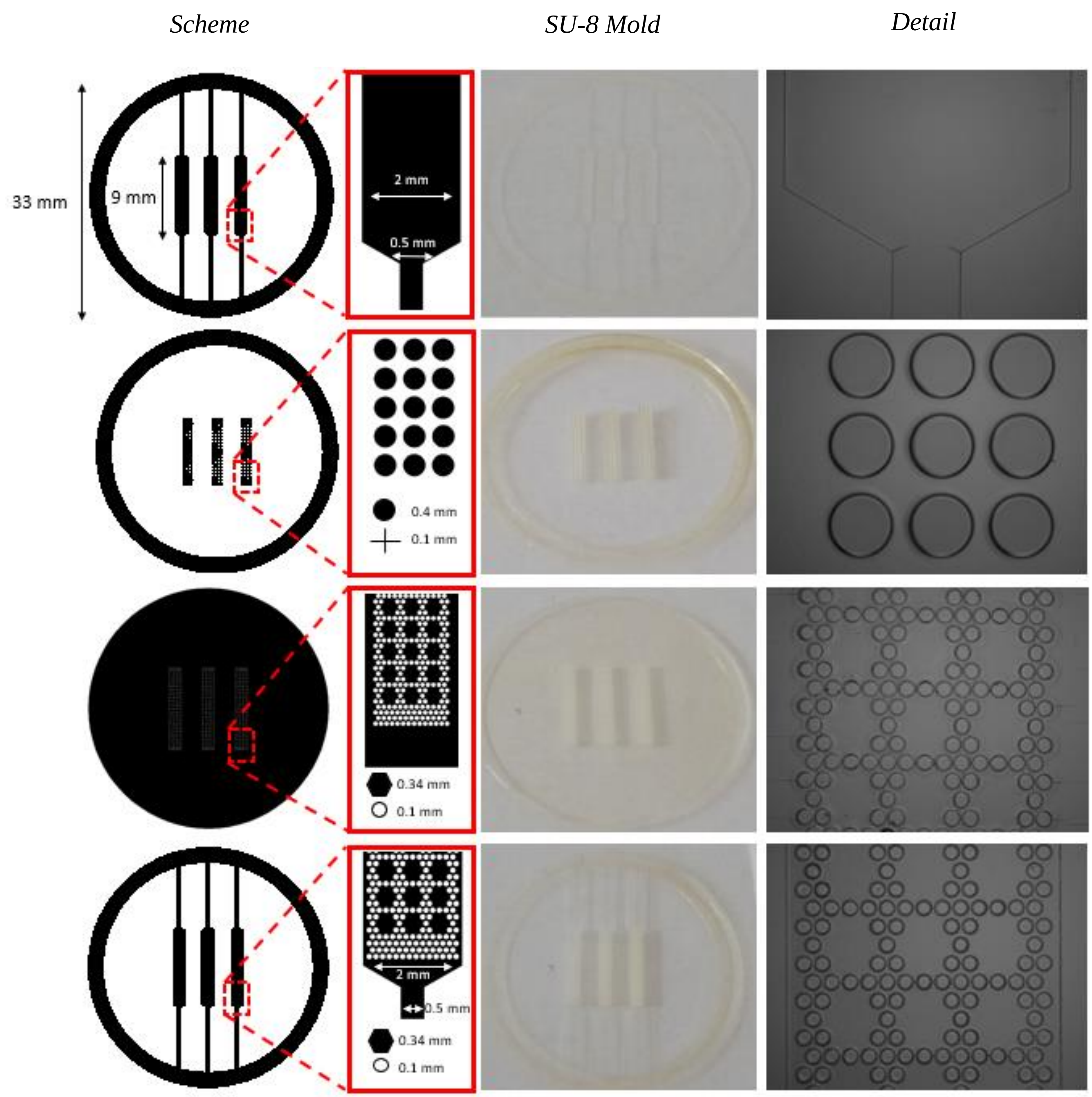

Schemes and photos of four different SU-8 molds (channel, microwell, micropillar, platform for microwell-pillar testing) for microfluidic platform preparation. Microfluidic platforms have the same sizes with different cultivation designs.

4. Tubing and connecting system

- Silicon tubing: 0.5 x 0.4 x 1.3 mm; 5.0 x 1.0 x 2.5 mm
- Teflon tubing: 0.8 x 1.6 x 0.4 mm
- Needles: 0.60 x 60 mm (23 G); 0.9 x 40 mm (20 G)
- Biopsy puncher 0.5 mm

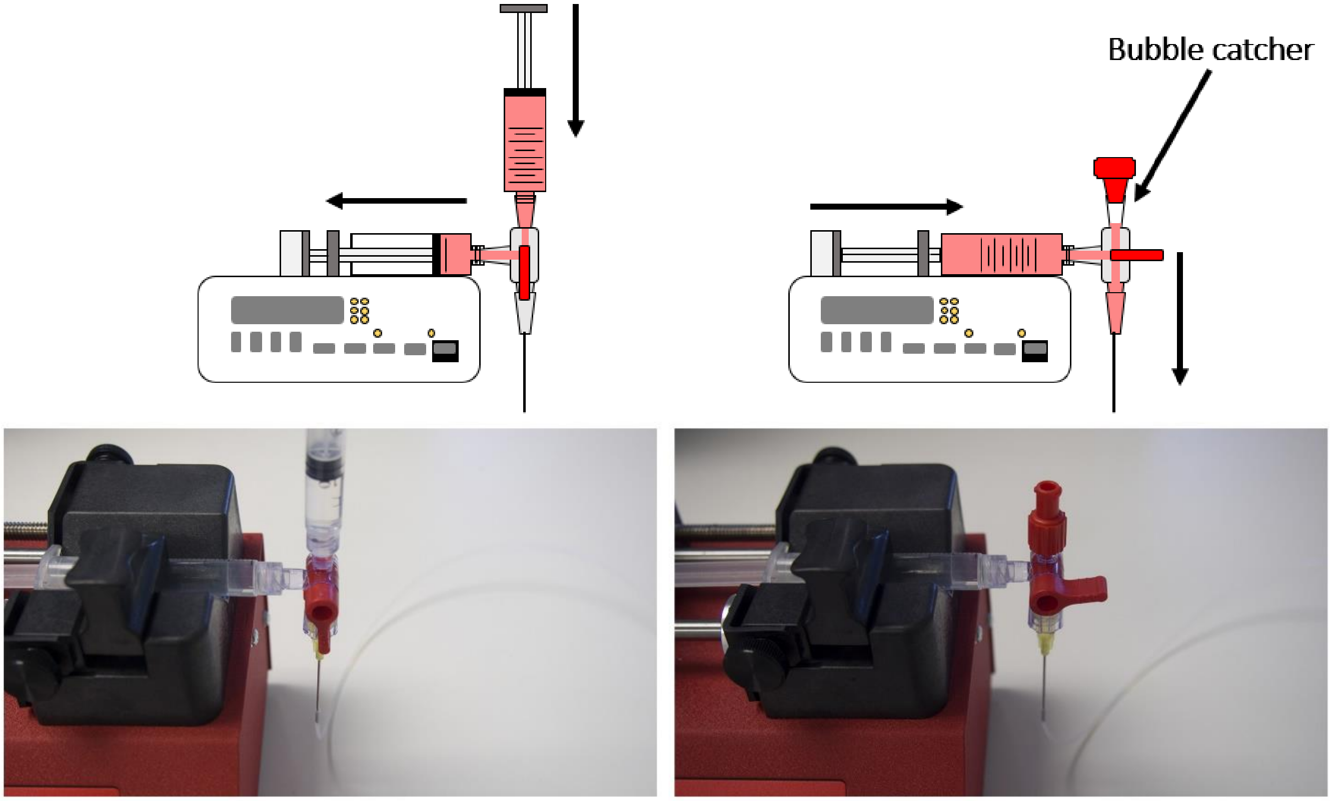

Media filling takes place through a three-way valve. Gas bubbles introduced into the system accumulate in the bubble capture space above the filling pipe that goes into the microfluidic system.

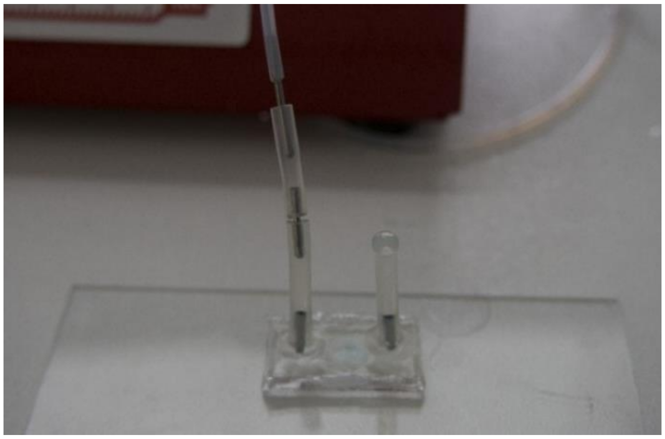

The tubes were connected via cut and polished needles. The silicon tubes and PDMS platform at the interface were modified by oxygen plasma for 60 sec, and a small amount of PDMS was added to eliminate the leaks of media and bubble formation.

5. Mobile Platforms Fabrication

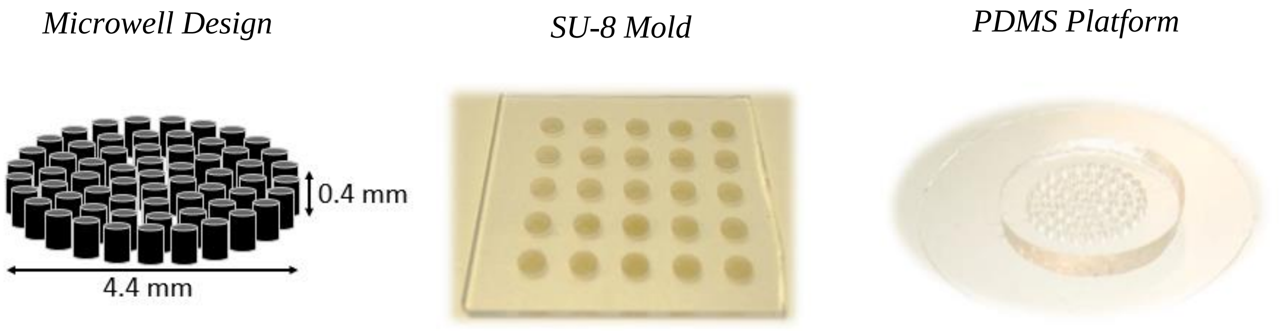

The design and fabrication of microwell platforms for static cultivation. Each platform was cut out by a corkscrew. The platforms were anchored to glass using oxygen plasma treatment for 40 seconds. After binding to glass, the platforms were placed on a heat plate (95 °C) for 20 min. The freshly prepared platforms can also be anchored to Petri dishes without plasma treatment. Before cell seeding, the platforms were degassed using cold alcohol, rinsed with distilled water, and coated with Pluronic F-127 (1:200) for 20 min. The degassing process can also be achieved using the vacuum system. The cells were seeded on the surface of the platforms in 30 µl spots, and after 60 min of incubator cultivation, they were kindly overlaid by media. The bone spheroids were observed after 12 hours of cultivation.

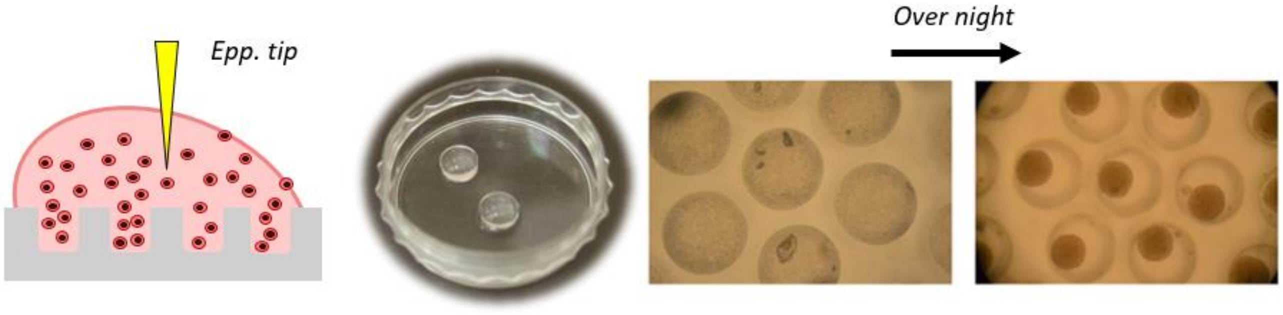

Closed microwell and microwell-pillar platform. The closed platforms were prepared in the same design as open microwell platforms. The microwell chip is composed of 64 wells with a diameter of 400 µm and a depth of 400 µm. The microwell-pillar platform consists of 64 wells with a diameter of 340 µm and a depth of 30 µm. The pillars have a diameter of 100 µm and a height of 200 µm. The bone cells cultivated in these mobile platforms can form spheroids.

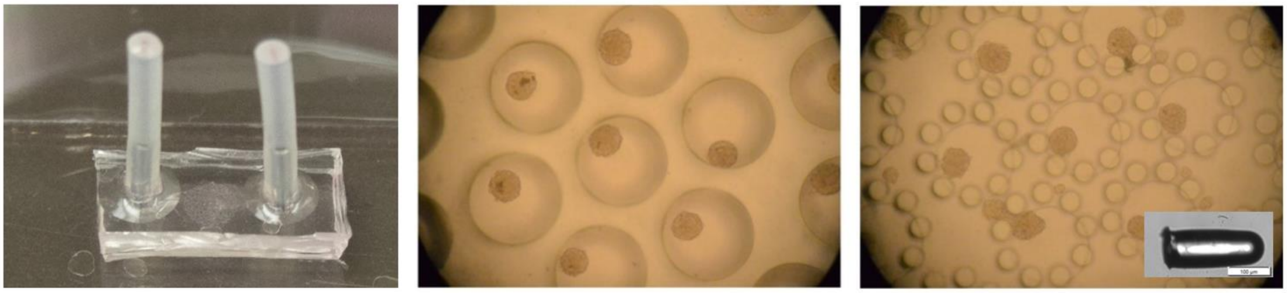

To compare the velocities of the designed platforms we used 0.4% Trypan Blue (Gibco, USA) for the media flow visualization. Time-lapse images were taken with the media flow rate of 2.5 µl/min, and the color spreading was analysed using ImageJ software. The linear platforms exhibited a higher flow rate than the mobile platforms. This fact corresponds to the smaller width of linear channels (2 mm) compared to circular platforms (4.6 mm). The flow velocity also decreased with the order from channel, to microwell and micropillar design. The micropillars form the obstruction for the media flow thus altering the velocity of individual springs.

**Type of the platform**	**Channel width (µm)**	**Microwell height (µm)**	**Micropillar height (µm)**	**Average measured velocity**
Linear channel	2000	N/A	N/A	264 µm/s
Linear wells	2000	400	N/A	210 µm/s
Linear well-pillars	2000	N/A	200	178 µm/s
Mobile wells	4600	400	N/A	100 µm/s
Mobile well-pillars	4600	30	200	83 µm/s

6. 3D Cultures in Bone Regeneration

Spheroids cocultivated in Matrigel next to each other exhibited the potential to fuse. Moreover, the cocultivation of murine MC3T3-E1 spheroids with fragments of the bones led to the continuous fusion of spheroids into the bone mass. The regeneration potential also displayed simple cultivation of bone spheroids on the surface of the bone.

Hanging drops spheroid attraction to bone fragment. (A) MC3T3-E1 spheroids mounted in Matrigel (A, G). The 3D spheroid mass continuously grew into the bone fragment during cultivation.

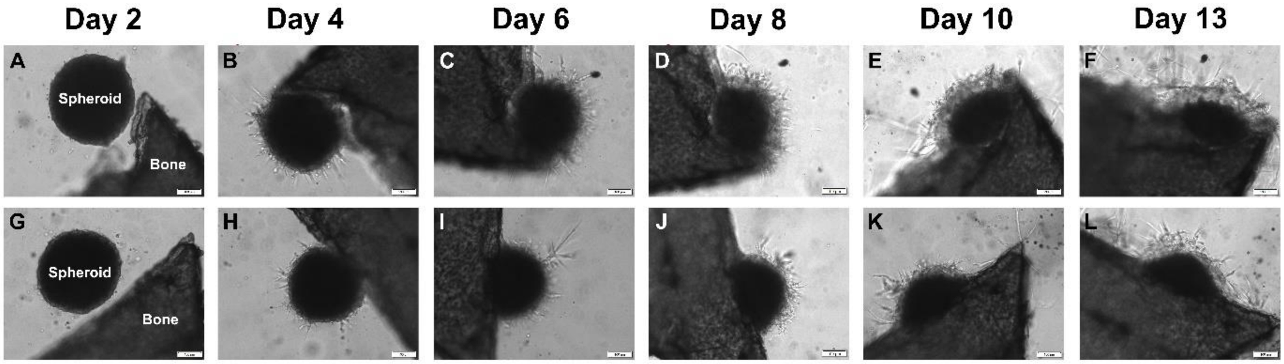

Bone defect filling by MC3T3-E1 bone spheroids prepared by the hanging drop system. Drilled hollows were covered by 2-day spheroids and cultivated for another 10 days. A, B, and C represent different bone fragments. The arrows indicate the hollows filled with tiny spheroids after 10 days of cultivation. The mineralized matrix was visualized by Alizarin red staining

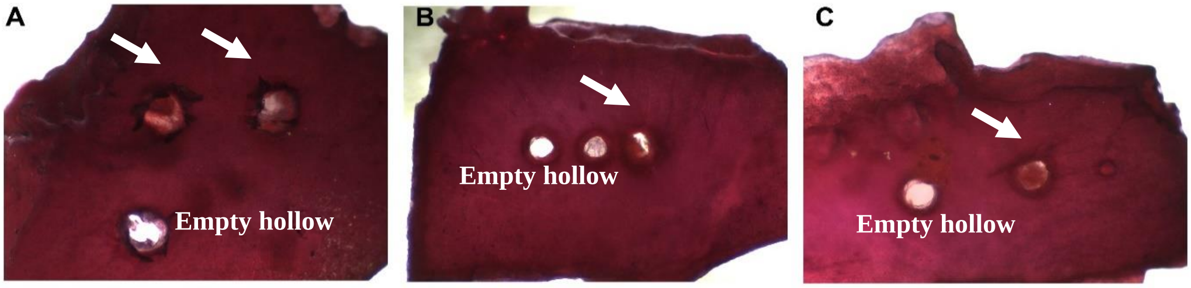

Supplement: Supplementary file 1 — Additional file 1. [file 13036_2023_395_MOESM1_ESM.pdf]
